# Phytochemical Profiling, In Vitro Bioactivity, and Network Pharmacology of *Astragalus cruciatus* Link Ethanolic Extract: Multitarget Mechanisms Underlying Potential Antidiabetic Effects

**DOI:** 10.3390/molecules31162831

**Published:** 2026-08-13

**Authors:** Leila Bellebcir, Imene Derardja, Redouane Rebai, Luc Jasmin, Abdennacer Boudah

**Affiliations:** 1 Laboratory of Genetics, Biotechnology and Bio-Resources Valorization, Department of Biology, Faculty of Exact Sciences and Natural Life Sciences, Mohamed Khider University, P.O. Box 145 RP, Biskra 07000, Algeria; 2 Department of Biology, Faculty of Exact Sciences and Natural Life Sciences, Mohamed Khider University, P.O. Box 145 RP, Biskra 07000, Algeria; 3 Department of Oral and Maxillofacial Surgery, University of California, San Francisco, 707 Parnassus Ave Suite D-1201, San Francisco, CA 94143, USA; 4 National Higher School of Biotechnology, Constantine 25000, Algeria

**Keywords:** *Astragalus cruciatus* Link, antidiabetic effect, T2DM, α-amylase inhibition, α-glucosidase inhibition, network pharmacology

## Abstract

Astragalus species have long been recognized for their pharmacological relevance, yet the antidiabetic properties of *Astragalus cruciatus* Link (Ac) remain poorly explored. This study aimed to investigate the antidiabetic potential of *A. cruciatus* Link and to elucidate possible underlying mechanisms. The ethanolic extract of Ac (AcEE) was prepared and analyzed for phenolic and flavonoid content. Antioxidant activity was assessed through a series of in vitro assays. The in vitro inhibition of α-amylase and α-glucosidase was assessed, followed by molecular docking to probe ligand-enzyme interactions. LC-ESI-MS analysis revealed a polyphenol-rich profile, with rutin (953.54 µg/g extract) and quinic acid (797.18 µg/g of extract) identified as main compounds. The AcEE showed significant inhibitory activity against both α-amylase and α-glucosidase (IC_50_ = 239.30 ± 7.40 and 192.60 ± 15.51 μg/mL, respectively). Moreover, low cytotoxic effects were observed in hepatic cell lines. Computational analysis revealed stable interactions between rutin and both enzymes (−12.935 and −8.073 Kcal/mol, respectively). Network pharmacology revealed that AcEE may modulate key targets, including IL-6, TNF-α, IL-1β, Akt-1, STAT3, EGFR, and INSR as well as pathways related to insulin resistance, AGE-RAGE, and inflammation. These findings suggest that AcEE exerts significant antidiabetic effects through multitarget modulation, highlighting its potential as a natural therapeutic agent for T2DM.

## 1. Introduction

Diabetes mellitus (DM) continues to impose an unprecedented burden on global health, with its prevalence rising steadily over the past few decades, making it one of the most pressing public health challenges of the 21st century, despite significant advances in disease prevention and clinical management [1]. According to the International Diabetes Federation, approximately 537 million people worldwide currently live with diabetes, a number expected to reach 700 million by 2045 [2]. Characterized by persistent hyperglycemia resulting from impaired insulin secretion, insulin action, or both, diabetes is associated with progressive metabolic dysfunction and is considered a primary cause of cardiovascular disease, blindness, kidney failure, and lower-limb amputation [1].

Type 2 diabetes mellitus (T2DM), accounting for nearly 90–95% of diabetic patients, is a complex and chronic metabolic disease characterized by insulin resistance, progressive β-cell dysfunction, chronic low-grade inflammation, and dysregulated glucose and lipid metabolism. These pathological processes are orchestrated through interconnected signaling networks, including phosphatidylinositol-3-kinase/protein kinase B (PI3K/Akt), AMP-activated protein kinase (AMPK), mitogen-activated protein kinase (MAPK), and peroxisome proliferator-activated receptor (PPAR), which collectively regulate insulin signaling, glucose uptake, energy homeostasis, lipid metabolism, and inflammatory responses [3]. Consequently, therapeutic strategies directed toward a single molecular target often fail to adequately address the multifactorial nature of T2DM, highlighting the need for therapeutic agents capable of simultaneously modulating multiple pathogenic pathways.

Current pharmacological management of T2DM relies on several classes of glucose-lowering agents, including biguanides, thiazolidinediones, dipeptidyl peptidase-4 inhibitors, glucagon-like peptide-1 receptor agonists, and insulin therapy. Although these medications have substantially improved glycemic control and reduced diabetes-related complications, their efficacy may decline over time as the disease progresses, while adverse effects, contraindications in specific patient populations, and the economic burden associated with lifelong therapy continue to limit optimal disease management [4]. Among the established therapeutic strategies for controlling postprandial hyperglycemia, inhibition of the carbohydrate-hydrolyzing enzymes, α-amylase and α-glucosidase, has proven particularly effective. These enzymes play essential roles in the digestion of dietary carbohydrates into absorbable monosaccharides; therefore, their inhibition delays glucose absorption and attenuates postprandial blood glucose excursions. Clinically approved inhibitors such as acarbose, miglitol, and voglibose effectively reduce postprandial hyperglycemia but are frequently associated with gastrointestinal adverse effects, including abdominal discomfort, flatulence, and diarrhea. These limitations have encouraged the search for naturally derived enzyme inhibitors with improved safety profiles and broader pharmacological relevance [5].

Beyond impaired glucose metabolism, chronic low-grade inflammation and oxidative stress are now recognized as central contributors to insulin resistance and disease progression. Dysfunctional adipose tissue acts as an active endocrine and immunological organ by secreting adipokines and pro-inflammatory cytokines that regulate metabolic homeostasis. During obesity and T2DM, dysregulation of adipose tissue results in excessive production of inflammatory mediators, particularly tumor necrosis factor-alpha (TNF-α), interleukin-6 (IL-6), and interleukin-1 beta (IL-1β), which interfere with insulin signaling and promote β-cell dysfunction, endothelial injury, and diabetic complications [6,7]. Consequently, therapeutic strategies capable of simultaneously attenuating oxidative stress, inflammation, and postprandial hyperglycemia have attracted increasing attention for the management of T2DM.

Plant-derived extracts are increasingly considered effective alternatives to synthetic drugs due to their high content of bioactive metabolites, including polyphenols, flavonoids, tannins, and carotenoids [6,7]. These compounds exhibit strong radical-scavenging activity, showing hypoglycemic effects with low toxicity and few adverse reactions, and confer multiple health benefits, including anti-inflammatory, anticancer [8,9,10], and antidiabetic effects, particularly bioactive constituents from the Fabaceae and Liliaceae families [11,12]. Their multitarget and synergistic mechanisms contribute to the mitigation of oxidative stress and related pathological conditions, while reducing the risk of adverse effects associated with synthetic drugs.

The genus *Astragalus* (Fabaceae), comprising more than 2500 species distributed worldwide, represents one of the richest botanical sources of bioactive secondary metabolites.

These predominantly ligneous species play a crucial role in mitigating desertification and are traditionally used as forage plants, ornamentals, and medicinal resources in local folk medicine [13,14,15]. Within the North African flora, particularly in the Saharan and Mediterranean regions, approximately 10 species of the genus *Astragalus* (Fabaceae) are endemic to Algeria, Morocco, and Tunisia [13,16], and 15 species are present in the Algerian Sahara. Among these species, a rare and noteworthy plant is *Astragalus cruciatus* Link, known locally as “Bouakifa” [17]. This species is also known under four synonyms: “*A. aristidis* Coss.”, “*A. radiatus* Ehrenb.”, “*A. trabutianus* Batt.”, and *A. corrugates* Bertol [18].

Algerian Saharan *Astragalus* species constitute a valuable source of bioactive secondary metabolites, particularly polysaccharides, flavonoids (including isorhamnetin glycosides), and saponins, which are associated with diverse pharmacological activities such as antioxidant, hepatoprotective, anti-inflammatory, immunostimulatory, antiviral, antidiabetic, and cytotoxic effects [19,20,21,22,23,24,25,26,27,28,29]. These bioactivities support their traditional use in the management of ailments such as nephritis, diabetes, leukemia, and envenomation.

In contrast to the extensive body of research devoted to other members of the genus, *Astragalus cruciatus* Link remains one of the least investigated *Astragalus* species from both phytochemical and pharmacological perspectives. To date, the available evidence is confined to a single phytochemical investigation describing its chemical composition [19] and a recent study demonstrating the antihyperglycemic potential of a galactomannan-rich seed mucilage through α-amylase and α-glucosidase inhibition [30]. While these findings provide an important foundation, they neither reflect the biological activity of the whole ethanolic extract nor identify the phytochemical constituents responsible for its effects. Consequently, the antidiabetic efficacy of the ethanolic extract, its active compounds, their molecular targets, and the molecular mechanisms underlying its pharmacological activity remain unknown. Moreover, no previous study has integrated experimental enzyme inhibition assays with network pharmacology and advanced molecular modeling techniques to comprehensively characterize the multitarget antidiabetic potential of this species.

Given the multifactorial nature of T2DM and the chemical complexity of medicinal plants, experimentally unraveling the molecular mechanisms underlying their therapeutic effects remains a considerable challenge. Recently, the integration of computational techniques with experimental pharmacology has emerged as a powerful and cost-effective strategy for natural product research. By enabling the prioritization of bioactive compounds, prediction of therapeutic targets, and exploration of complex molecular interaction networks, these approaches accelerate hypothesis generation and provide mechanistic insights that guide and complement experimental investigations [1]. Among these approaches, network pharmacology offers a systems-level perspective of the interactions between phytochemicals, molecular targets, and disease-associated pathways, which help identify key regulatory nodes and pathways affected by natural compounds. This facilitates evaluating the potential of plant extracts for therapeutic use and identifying novel molecular targets [31]. Molecular docking complements this by predicting how bioactive compounds bind to target proteins, whereas MM/GBSA calculations refine binding free-energy estimations and molecular dynamics (MD) simulations evaluate the stability and dynamic behavior of protein–ligand complexes under near-physiological conditions.

Accordingly, the present study aimed to comprehensively evaluate the antidiabetic potential of the ethanolic extract of *Astragalus cruciatus* Link through in vitro α-amylase and α-glucosidase inhibitory assays. To gain mechanistic insight into the observed biological activity, an integrated computational framework combining network pharmacology, molecular docking, induced-fit docking (IFD), MM/GBSA binding free-energy calculations, and molecular dynamics (MD) simulations was employed to identify putative therapeutic targets, characterize protein–ligand interactions, and generate mechanistic hypotheses underlying the multitarget antidiabetic effects of the extract.

## 2. Results

### 2.1. Extraction Yield and Phytochemical Composition

The extraction yield, total phenolic and total flavonoid contents of AcEE are summarized in Table 1. According to the results, AcEE presented an extraction yield of 13.50% (Table 1). Phytochemical quantification revealed a total phenolic content of 186 ± 0.20 mg GAE/g DW and a total flavonoid content of 45.63 ± 0.04 mg QE/g DW (Table 1).

To further characterize the phytochemical profile, LC-ESI-MS analysis was performed using 35 reference standards encompassing phenolic acids and flavonoids. As a result, 19 compounds were identified in AcEE, including 10 phenolic acids and 9 flavonoids (Table 2).

Among phenolic acids, quinic acid was the predominant compound (797.186 µg/g of extract), followed by p-coumaric acid (66.329 µg/g of extract), protocatechuic acid (41.435 µg/g of extract), and trans-cinnamic acid (38.401 µg/g of extract). Flavonoids were mainly represented by flavones and flavonols, presenting as both glycosides and aglycones. Rutin and hyperoside were the predominant glycosylated flavonoids, whereas apigenin, luteolin, cirsiliol, cirsilineol, kaempferol, and naringenin were detected as aglycones (Table 2). Based on quantitative LC-ESI-MS data, rutin was the most abundant flavonoid in AcEE (953.542 µg/g of extract), followed by cirsiliol (81.845 µg/g of extract) and hyperoside (47.003 µg/g of extract) (Table 2).

### 2.2. In Vitro Antioxidant Activity

The antioxidant potential of AcEE was evaluated using multiple complementary assays, and the results are presented in Table 3. In the β-carotene-linoleic acid bleaching test, AcEE inhibited lipid peroxidation with an IC_50_ value of 1.849 ± 0.017 mg/mL, whereas BHA showed an IC_50_ value of 0.267 ± 0.014 mg/mL. In the ferrous ion chelating assay, AcEE showed a chelating capacity with an IC_50_ of 1.382 ± 0.017 mg/mL, which was weaker than that of EDTA (IC_50_ = 0.051 ± 0.001 mg/mL).

On the other hand, the results of the DPPH assay revealed that AcEE presented comparatively notable radical scavenging activity. The extract showed a significant ability to scavenge DPPH radical with an IC_50_ of 0.062 ± 0.003 mg/mL, although it was approximately two-fold less potent than BHA (IC_50_ = 0.032 ± 0.002 mg/mL) (Table 3). Additionally, in the FRAP assay AcEE exhibited significant ferric-reducing capacity with an IC_50_ value of 0.267 ± 0.012 mg/mL, approximately 2.7-fold higher than that of BHA (IC_50_ = 0.100 ± 0.007 mg/mL) (Table 3). The total antioxidant capacity determined by the phosphomolybdate method was 26.48 ± 0.38 mg GAE/g DW (Table 3), suggesting significant activity.

### 2.3. In Vitro Alpha-Amylase and Alpha-Glucosidase Inhibition

The inhibitory activities of Ac extract against α-amylase and α-glucosidase are summarized in Table 4. The results revealed a clear concentration-dependent inhibition for both enzymes, with increasing inhibitory activity observed at higher extract concentrations. Against α-amylase, AcEE exhibited an IC_50_ value of 239.30 ± 7.40 µg/mL, indicating a notable inhibitory effect. Similarly, AcEE demonstrated a significant inhibitory effect toward α-glucosidase, with an IC_50_ value of 192.60 ± 15.51 μg/mL. Overall, the extract exhibited a more pronounced inhibitory effect on α-glucosidase compared to α-amylase. In comparison, the standard drug, acarbose, showed IC_50_ values of 94.78 ± 8.48 µg/mL for α-amylase and 211.70 ± 17.09 µg/mL for α-glucosidase.

These results suggest that the extract exhibited a comparatively greater inhibitory effect against α-glucosidase than the reference drug, while showing lower inhibitory activity against α-amylase.

### 2.4. Cytotoxicity Assessment

The results of the MTT cell viability test are presented in Table 5 and Figure S1. Compared with the untreated cells, AcEE produced concentration-dependent effects on cell viability in both AML12 and HepG2 cells, with low cytotoxicity at concentrations up to 250 μg/mL (Figure S1). The extract showed an inhibitory effect on cell viability, with IC_50_ values of 403.40 ± 23.26 and 396.30 ± 44.33 μg/mL for AML12 cells and HepG2 cells, respectively (Table 5). In contrast, the reference anticancer agent cisplatin displayed substantially higher cytotoxicity on both AML12 and HepG2 cell lines, with IC_50_ values of 8.34 ± 0.42 μg/mL and 5.95 ± 0.60 μg/mL, respectively (Table 5).

Hence, our findings indicated that the AcEE had low cytotoxicity against the HepG2 and AML12 cell lines, particularly at concentrations required for α-amylase and α-glucosidase inhibition.

### 2.5. In Silico Analysis of Enzyme-Ligand Interactions

#### 2.5.1. Molecular Docking and MM/GBSA Findings

As an initial validation step, the root-mean-square-deviation (RMSD) between the crystallographic pose of the native inhibitor and the re-docked conformation within the α-amylase active site was calculated. The obtained RMSD value of 0.638 Å confirmed the reliability of the docking procedure (Figure S2). Subsequent docking simulations revealed noticeable differences in the binding tendencies of AcEE’s constituents toward the targeted enzymes. Overall, flavonoid derivatives consistently displayed the strongest binding tendencies, with rutin emerging as the top-ranked compound in terms of predicted binding affinity. In contrast, simpler phenolic acids showed comparatively weaker binding affinities. The detailed binding score values obtained for all screened compounds are presented in Table 6.

Against α-amylase, rutin achieved a Glide score of −12.935 Kcal/mol, markedly outperforming the reference drug (−9.364 Kcal/mol), while its affinity for α-glucosidase (−8.073 Kcal/mol) was comparable to that of acarbose (−8.902 Kcal/mol). Other flavonoids, including quercetin-3-O-galactoside, luteolin, kaempherol, and apigenin, displayed comparatively moderate but significant affinities against both enzymes (Table 6).

To further reinforce the docking results, the binding free energies (ΔG_bind) of the docked complexes were estimated using the MM/GBSA approach (Table 6). Consistent with the docking predictions, rutin exhibited the most favorable binding free energy toward α-amylase (−68.53 Kcal/mol), surpassing the reference inhibitor acarbose (−62.02 kcal/mol), while quercetin-3-O-galactoside (−59.63 Kcal/mol) and luteolin (−51.92 Kcal/mol) also showed favorable binding energies. For α-glucosidase, acarbose displayed the lowest binding free energy (−58.60 Kcal/mol), followed closely by rutin (−54.80 Kcal/mol). Notably, apigenin (−52.06 Kcal/mol), apigenin-7-O-glucoside (−52.04 Kcal/mol), and naringenin (−50.62 Kcal/mol) exhibited favorable MM/GBSA energies despite their comparatively moderate docking scores, suggesting stable protein–ligand complexes. These findings indicate that the high docking affinity of rutin is supported by a favorable energetic profile, reinforcing its potential as a dual α-amylase/α-glucosidase inhibitor. However, its concentration in the extract is substantially low than the reported values for pure rutin [32], suggesting that rutin alone does not account for the observed in vitro inhibitory activity of the extract.

Interactions analysis indicated that rutin was stably accommodated in both binding sites through several contacts, where its flavonoid core and glycosidic moiety contributed distinctly to complex stabilization. In the α-amylase active pocket, the hydroxyl-rich flavonoid core formed hydrogen bonds with Trp59, Gln63, and Gly306, while the glycosidic moiety reinforced binding via additional interactions with Lys200, His201, Asp300, and Glu233 (Figure S3). Beyond hydrogen bonding, rutin established π-π stacking interactions with Trp59 and His305, alongside multiple hydrophobic contacts involving Trp58, Tyr62, Tyr151, and Leu162, which collectively enhanced complex stability. In comparison, acarbose binding was mainly stabilized by hydrogen bonds with Glu233, His305, and Gly306, supported by hydrophobic contacts with Trp58, Trp59, Tyr62, and Val98 (Figure S4).

With α-glucosidase, rutin similarly demonstrated a stable binding mode, with the flavonoid core forming hydrogen bonds with Gln353, Glu411, and Arg442, and its sugar moiety extended into the enzyme’s pocket, establishing hydrogen bonds with Leu313 and Glu411. Additional stabilization was conferred by stacking interactions between the aromatic core and Tyr72, as well as by several hydrophobic contacts (Figure S5). In contrast, acarbose established hydrogen bonds with Tyr158, Gln279, Leu313, Gln353, and Arg442, supported by hydrophobic contacts with residues such as Phe159, Phe178, Val216, and Leu219 (Figure S6).

#### 2.5.2. Induced Fit Docking (IFD) Analysis

The induced-fit docking (IFD) analysis corroborated the initial docking results by revealing comparable affinities of the top compound for α-amylase and α-glucosidase. Additionally, IFD demonstrated enhanced interaction profiles for the rutin compound relative to the rigid receptor docking.

For the rutin–α-amylase complex, almost all ligand-protein interactions remained constant after IFD studies and showed the best IFD score of −1330.60 Kcal/mol. Furthermore, the IFD results indicated the formation of new hydrogen bonds with Tyr62, Asp197, and His299, but the hydrogen bonds with Trp59 and Gly306 were lost (Figure S7A). Rutin also exhibited strong affinity toward α-glucosidase with an IFD score of −1123.60 Kcal/mol and formed additional hydrogen bonds with the residues Lys156, Pro312, and Asp352; however, the hydrogen bond with Gln353 was lost (Figure S7B). Hydrophobic and other interactions remained unchanged compared with XP docking, thereby enhancing its stability within the α-amylase and α-glucosidase binding sites.

The reference inhibitor, acarbose, showed IFD scores of −1328.20 Kcal/mol and −1122.72 Kcal/mol against α-amylase and α-glucosidase, respectively, which are lower than those observed for rutin. Along with keeping its interaction patterns from XP docking, acarbose also formed new hydrogen bonds with Val163, Asp197, and Asp356 in α-amylase, as well as with Asp69, His112, Arg213, and Asp215 in α-glucosidase (Figure S7C,D).

These results show that the lead compound exhibits strong inhibition, suggesting it interacts more effectively with α-amylase and α-glucosidase.

#### 2.5.3. Molecular Dynamics Simulations

Given that docking provides a static representation of protein–ligand interactions, molecular dynamics simulations were performed to assess the dynamic stability of rutin in complex with α-amylase and α-glucosidase. Over a 100 ns trajectory, backbone RMSD, residue fluctuations (RMSF), and interaction persistence were monitored to gain deeper insights into the reliability of the predicted binding modes.

**Figure 1:**
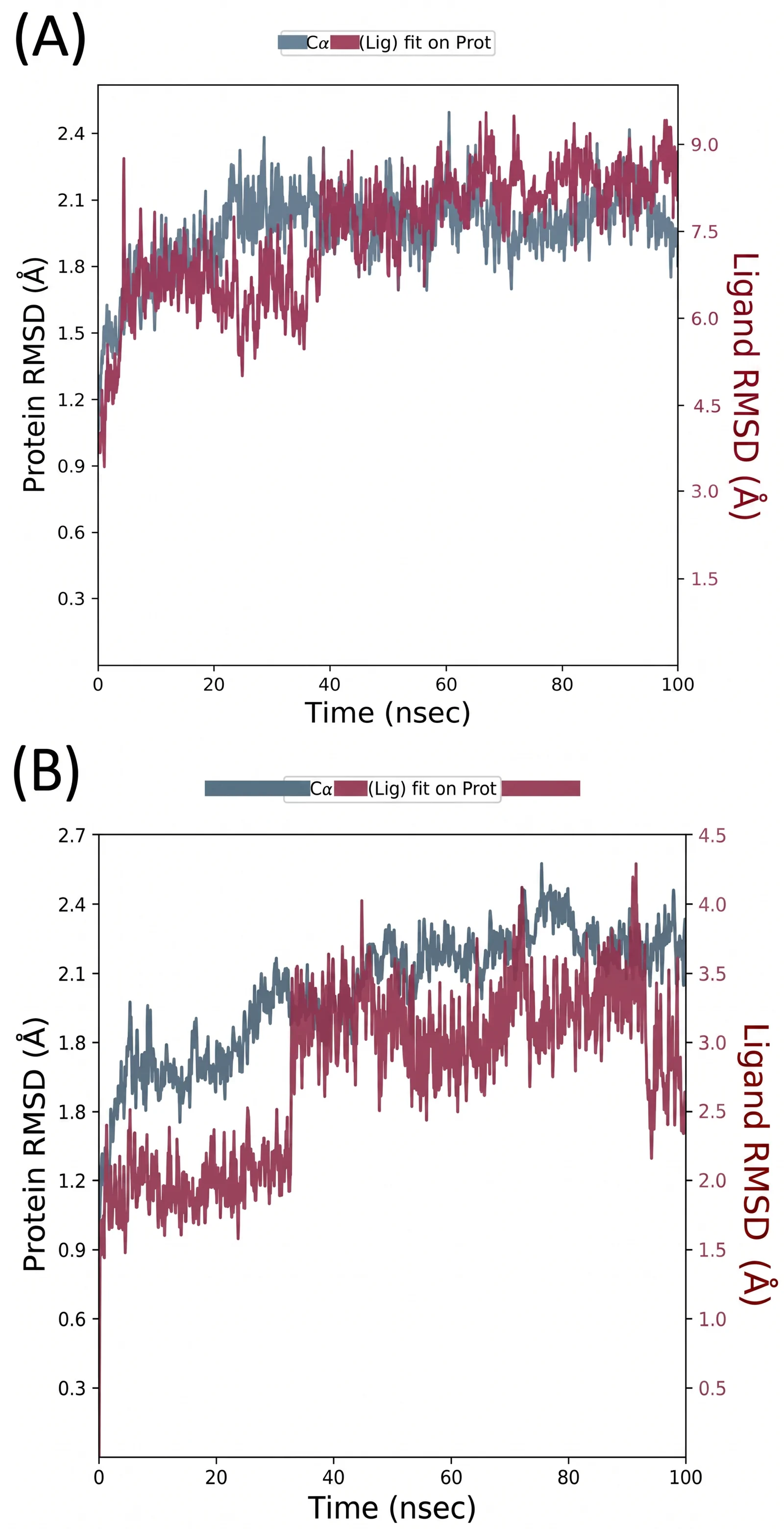
RMSD plots of top complexes over 100 ns MD simulation: (**A**) rutin-1OSE complex; (**B**) rutin-3A4A complex.

The RMSD analysis of the rutin–α-amylase complex revealed that the system reached equilibrium early in the simulation. The protein backbone RMSD increased to approximately 1.6–1.7 Å within the first 10 ns, reflecting structural relaxation from the initial conformation, and subsequently it stabilized, with fluctuations in the range of 1.9–2.3 Å for the remainder of the trajectory. Importantly, no significant deviations were observed after this stabilization, indicating that the enzyme’s global structural integrity was maintained throughout the 100 ns simulation period. In parallel, the ligand RMSD, calculated relative to the protein framework, increased initially over the first 30 ns, reflecting conformational adjustments and reorientation of rutin within the binding pocket. Following this phase, the ligand RMSD stabilized, fluctuating around an average of 7–9 Å until the end of the simulation (Figure 1A).

For the rutin–α-glucosidase complex, the RMSD profile similarly indicated stable dynamics over the 100 ns of simulation. The protein backbone RMSD showed a gradual increase during the initial equilibration period, reaching approximately 1.9 Å within the first 10 ns, followed by stabilization with fluctuations predominantly within the range of 2.0–2.4 Å. The ligand RMSD remained relatively low during the early stages of the simulation and increased after 40 ns, stabilizing thereafter at fluctuations around 3.5–4.0 Å. This behavior suggests that rutin remains stably associated with the α-glucosidase binding site while undergoing moderate conformational adaptation (Figure 1B).

**Figure 2:**
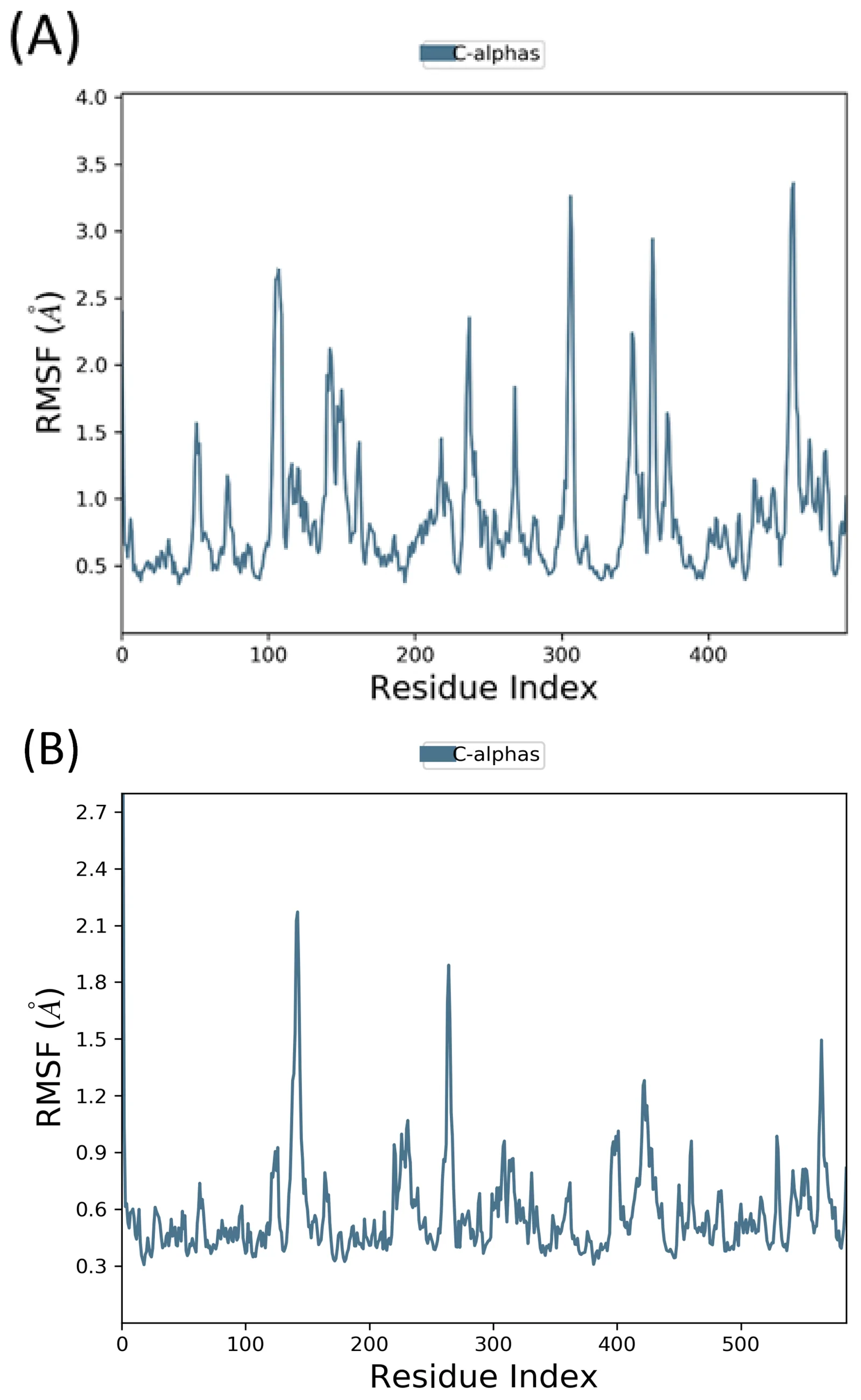
RMSF analysis of selected complexes over 100 ns MD simulation: (**A**) rutin-1OSE complex plot; (**B**) rutin-3A4A complex plot.

To gain further insight into residue-level flexibility and local dynamic behavior, root- mean-square-fluctuation (RMSF) analysis was performed for both complexes over the 100 ns molecular dynamics simulation. For the rutin–α-amylase complex, the RMSF profile revealed generally low fluctuations across the protein backbone, with the majority of residues exhibiting RMSF values not exceeding 3.0 Å (Figure 2A). Likewise, the rutin–α-glucosidase complex exhibited a more restricted fluctuation profile, with most residues displaying RMSF values below 2.5 Å throughout the simulation. Significant fluctuations were mainly observed in the N-terminal region, which is intrinsically flexible and not directly involved in ligand binding. In contrast, residues located in proximity to the binding pocket showed reduced mobility, supporting the persistence of protein–ligand interactions and a stable binding conformation (Figure 2B).

**Figure 3:**
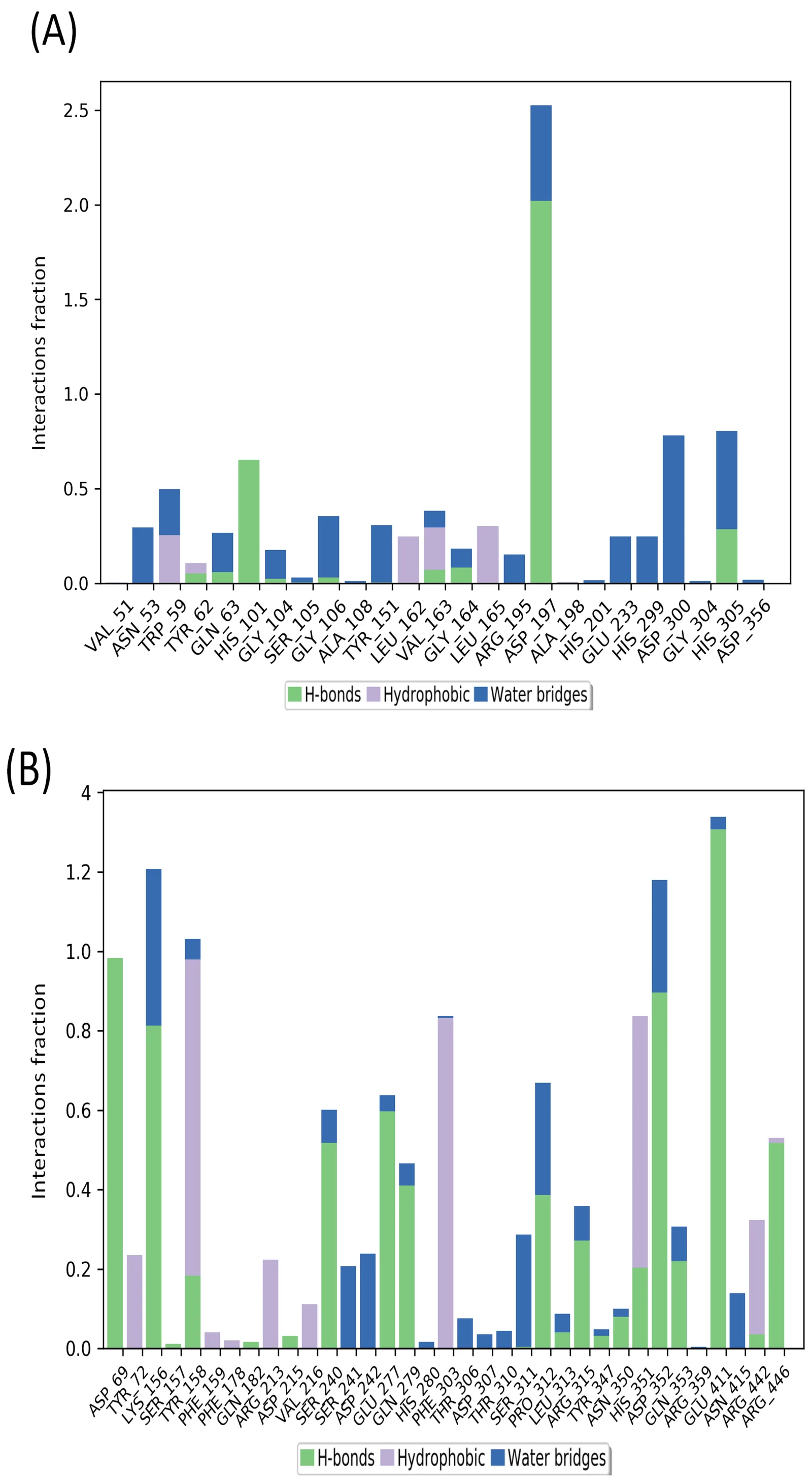
Histogram of: (**A**) the rutin–α-amylase complex; (**B**) the rutin–α-glucosidase complex during the 100 ns MD simulations.

The protein–ligand contact histograms corroborated RMSD and RMSF findings by demonstrating persistent hydrogen bonds, hydrophobic interactions, and water-mediated interactions between rutin and key active-site residues throughout the simulation (Figure 3A,B). The sustained occupancy of these contacts indicates that the docking-derived binding poses remained stable over the 100 ns trajectory for both the α-amylase and α-glucosidase complexes.

### 2.6. Network Pharmacology Findings

#### 2.6.1. Target Prediction and Intersection

Target mapping showed that the studied compounds are collectively associated with 1137 putative protein interactors, reflecting a broad interaction capacity within the human proteome and substantial pharmacological versatility. In parallel, a comprehensive type 2 diabetes mellitus (T2DM)-related gene set comprising 2920 genes was assembled by integrating 2228 genes from the GeneCards database and 840 genes from the OMIM database after duplicates were removed. Comparative analysis of these datasets identified 488 overlapping genes, corresponding to a pronounced molecular intersection through which AcEE compounds may modulate key regulatory components of T2DM pathophysiology.

#### 2.6.2. Protein–Protein Interaction (PPI) Network

**Figure 4:**
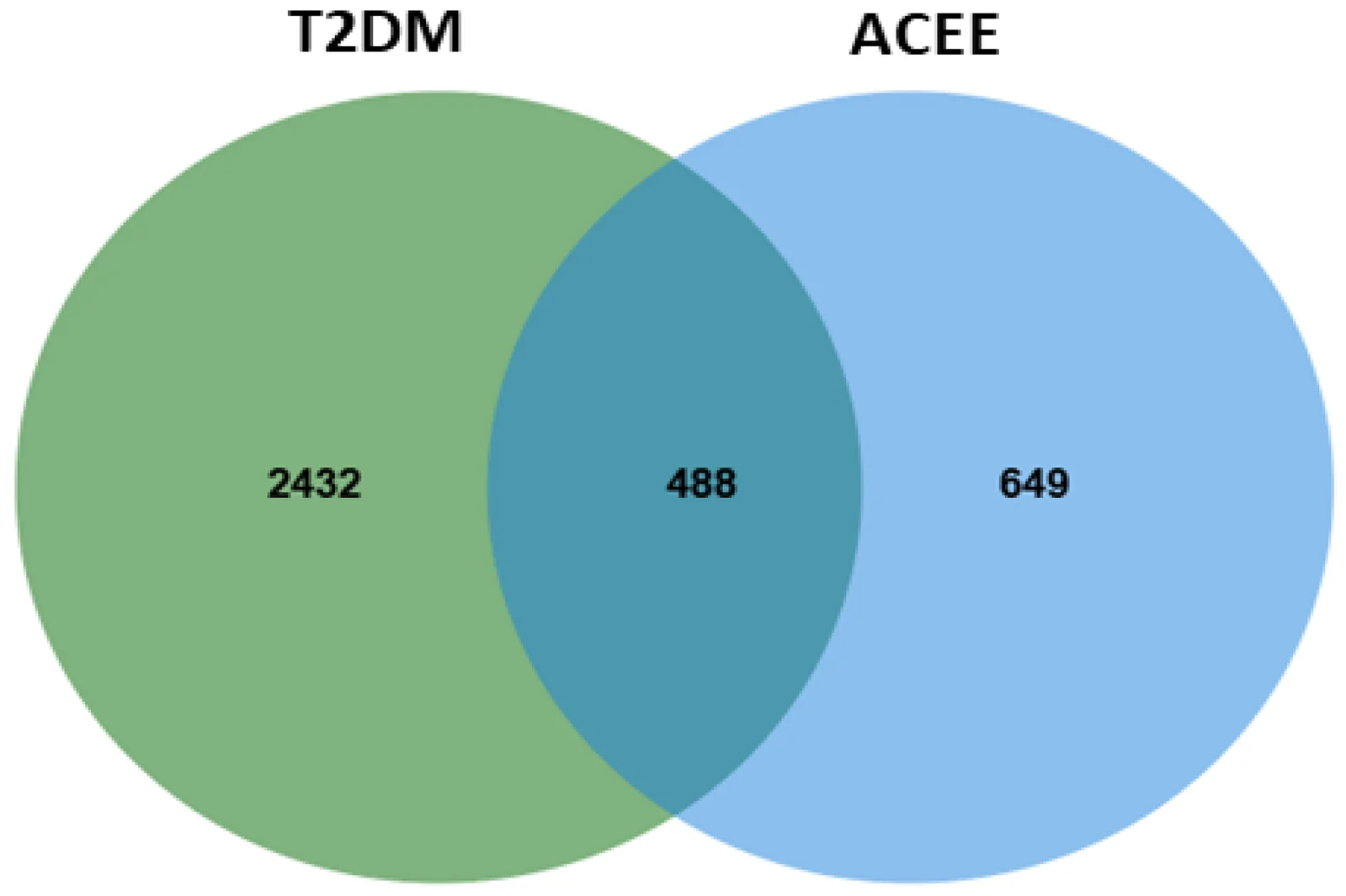
Venn diagram representation of the overlapping genes between AcEE compounds and type 2 diabetes.

To investigate the functional interplay among the 488 overlapping targets (Figure 4), a protein–protein interaction network was constructed. The resulting network comprised 455 nodes connected by 5018 edges. The network had an average node degree of 22.1 and a clustering coefficient of 0.474, indicating a densely connected system, where AcEE constituents are highly interrelated within T2DM pathways (Figure S8). Topological analysis of the PPI network highlighted a subset of core targets central to T2DM pathophysiology. Using multiple centrality measures, in particular, degree, closeness, betweenness, MNC, and EPC, the top 10 targets were ranked, and only those consistently identified across all algorithms were selected as hub genes. The intersection of these outcomes revealed that IL-6, TNF-α, IL-1β, Akt-1, STAT3, EGFR, and INSR are the most prominent regulatory nodes within the network (Figure S9).

#### 2.6.3. Diseases Targets-Functional Annotations Signaling Pathways Network

##### KEGG Pathways Enrichment Analysis

**Figure 5:**
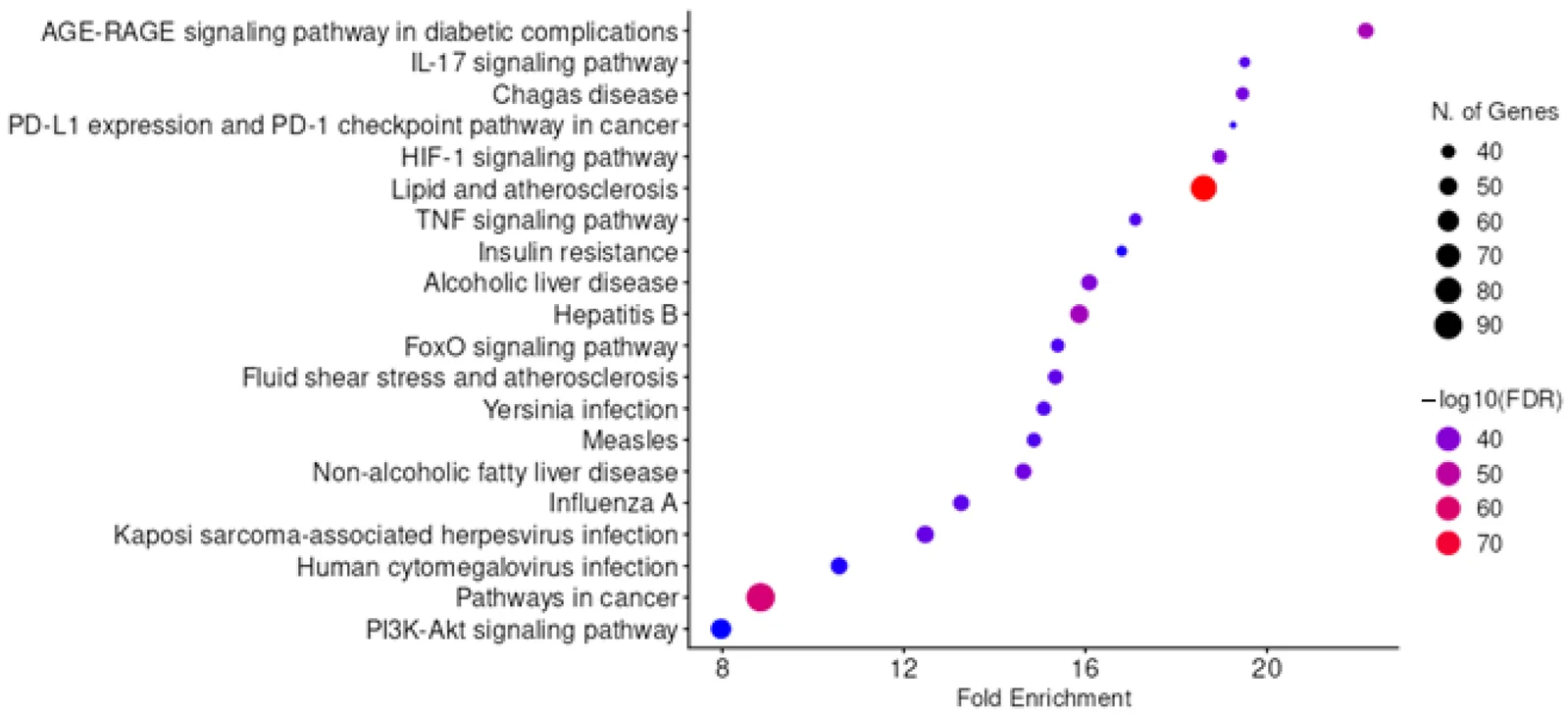
KEGG enrichment dot plot of the top 20 enriched pathways.

KEGG enrichment analysis of the 488 intersecting targets identified 282 significantly enriched pathways at a false discovery rate (FDR) < 0.01, from which the top 20 pathways were selected based on statistical significance and enrichment strength. As shown in Figure 5 the enriched pathways were predominantly associated with T2DM and its systemic complications, indicating significant disease relevance of the intersecting target set.

The most markedly enriched pathway was AGE-RAGE signaling in diabetic complications (FE = 23, 46 genes), followed by IL-17 (FE = 20.33, 37 genes), lipid and atherosclerosis (FE = 20.20, 85 genes), and insulin resistance (FE = 18, 39 genes) pathways. Likewise, inflammatory and stress-response pathways, including HIF-1 signaling (FE = 19, 42 genes) and TNF signaling (FE = 17, 41 genes), were significantly enriched among the top pathways. Metabolism-related pathways linked to hepatic dysfunction were also prominently represented, including alcoholic liver disease (FE = 16, 47 genes) and non-alcoholic fatty liver disease (FE = 14, 46 genes) (Figure 5). Pathways related to vascular biology were similarly enriched. Additionally, several immune and cancer-related pathways were detected, reflecting shared signaling cascades.

##### Gene Ontology (GO) Enrichment Analysis

**Figure 6:**
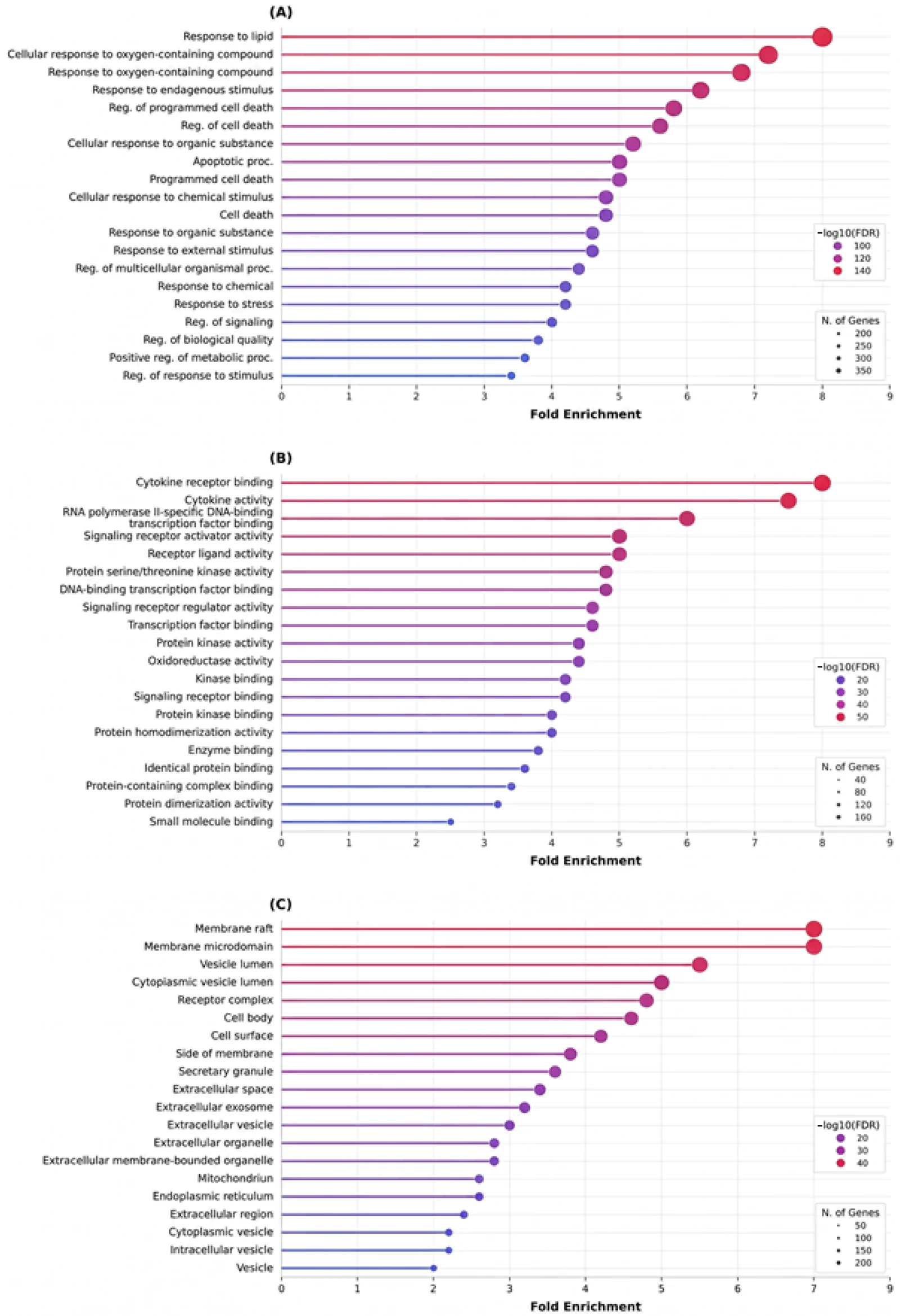
GO enrichment analysis of *Astragalus cruciatus* in the treatment of type 2 diabetes: (**A**) Lollipop of BP enrichment; (**B**) Lollipop of MF enrichment; **(C**) Lollipop of CC enrichment.

GO enrichment analysis of the intersecting genes identified a total of 1000 significantly enriched biological process (BP) terms, 932 molecular function (MF) terms, and 370 cellular component (CC) terms under the applied significance thresholds. For clarity and visualization, the top 20 enriched terms from each GO category were selected and are presented in Figure 6.

Within the BP category, response to lipid was the most significantly enriched term (FE = 8, 159 genes), followed by cellular response to oxygen-containing compound (FE = 7.4, 185 genes) and response to oxygen-containing compound (FE = 6.7, 234 genes). Additional marked enriched processes included cellular response to chemical stimulus (FE = 6.16, 289 genes), regulation of programmed cell death (FE = 4.8, 208 genes), cell death (FE = 4.8, 208 genes), apoptosis process (FE = 4.89, 203 genes), and response to stress (FE = 3.5, 319 genes). Terms related to positive regulation of signaling and positive regulation of cell communication were also represented among the main pathways (Figure 6A). In the MF category, cytokine receptor binding (FE = 7.80, 51 genes) and cytokine activity (FE = 7.08, 41 genes) ranked among the most significant terms. Other significant enriched pathways included RNA polymerase II-specific DNA-binding transcription factor binding (FE = 5.8, 43 genes), signaling receptor activator activity (RE = 5.51, 69 genes), receptor ligand activity (FE = 5.38, 66 genes), signaling receptor regulator activity (FE = 5.27, 71 genes), and transcription factor binding (FE = 4.89, 62 genes) (Figure 6B). Regarding the CC category, dominant localization was observed for membrane raft (6.21, 40 genes) and membrane micro-domain (FE = 6.17, 40 genes). Additional enriched components included vesicle lumen (FE = 5.73, 43 genes), cytoplasmic vesicle lumen (FE = 5.62, 42 genes), secretory granule lumen (FE = 5.42, 40 genes), receptor complex (FE = 5.19, 44 genes), and cell body (FE = 4.59, 53 genes) (Figure 6C).

##### Compound-Target-Pathway Network Construction

**Figure 7:**
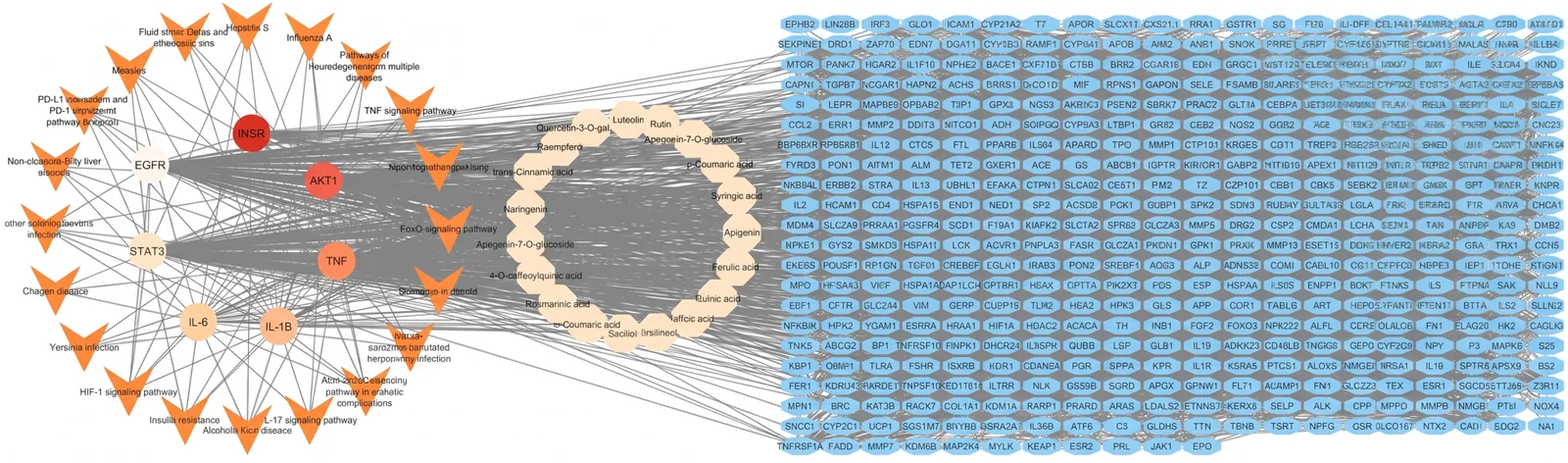
Compounds–targets–pathways network showing potential mechanism of AcEE for the treatment of type 2 diabetes.

Once the data on selective bioactive compounds, type 2 diabetes mellitus targets, and signaling pathways were prepared, they were imported into Cytoscape v.3.10.2 to create a compound-target-pathway network diagram. In Figure 7, circles show hub genes, V marks pathways, blue-purple ovals represent targets, and yellow-orange octagons stand for compounds.

#### 2.6.4. Molecular Docking Analysis

To assess the binding potential of the identified compounds toward key regulatory nodes within the PPI network, molecular docking was performed against seven core proteins: INSR (PDB ID: 5HHW), EGFR (PDB ID: 5HG8), Akt-1 (PDB ID: 1UNQ), STAT3 (PDB ID: 6NUQ), TNF-α (PDB ID: 2AZ5), IL-1β (PDB ID: 8RYK), and IL-6 (PDB ID: 5FUC).

Redocking of the co-crystallized or an appropriate reference inhibitor for each target yielded RMSD values below 2.0 Å (Table S1), confirming the reliability of the docking protocol. Validation procedure and subsequent comparative analysis were based on the co-crystallized ligands for INSR, EGFR, STAT3, and TNF-α. For Akt-1, IL-1β, and IL-6, where suitable co-crystallized small-molecule ligands were unavailable, well-characterized reference inhibitors were used only for comparative analyses.

**Figure 8:**
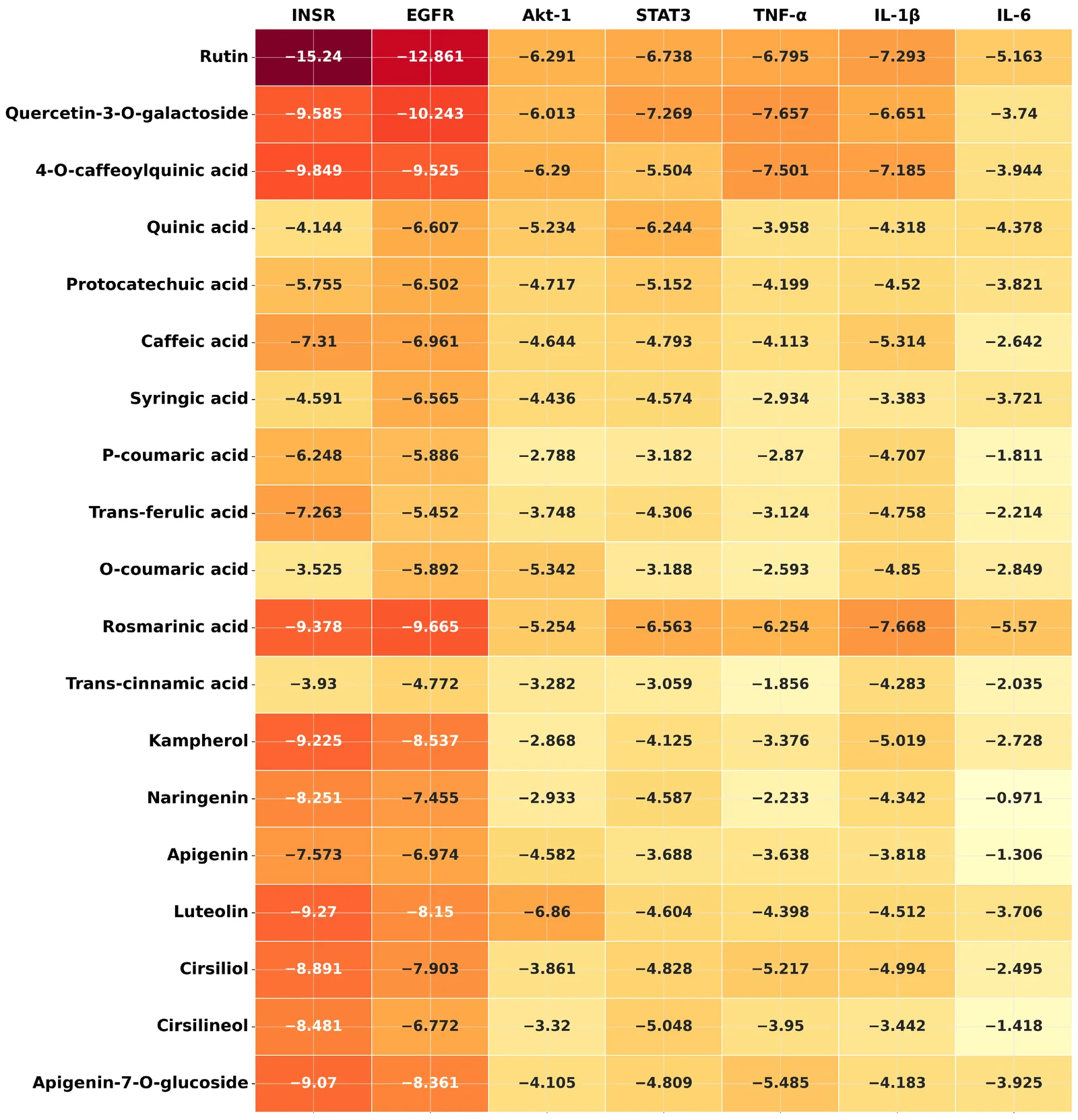
Heatmap representation of molecular docking affinities between AcEE constituents and the hub proteins.

Analysis of binding patterns revealed distinct target preference and compound selectivity profile, with binding energies ranging from −15.240 to −0.971 Kcal/mol (Figure 8). In particular, AcEE compounds showed notable binding for tyrosine kinase receptors (INSR and EGFR), followed by inflammatory cytokines and intracellular signaling proteins.

Docking against INSR revealed exceptionally strong binding affinities, with 13 compounds exhibiting binding energies exceeding −7 Kcal/mol. Among them, rutin emerged as the potent binder (−15.240 Kcal/mol), which represents the highest affinity recorded across the entire docking dataset. Additional compounds showing high affinity included 4-O-caffeoylquinic acid (−9.849 Kcal/mol), quercetin-3-O-galactoside (−9.585 Kcal/mol), and rosmarinic acid (−9.378 Kcal/mol). Notably, the predicted docking score of rutin was more favorable than that of the co-crystallized inhibitor (−11.880 kcal/mol) (Table S1).

Similarly, EGFR displayed strong ligand binding, with 9 compounds displaying binding energies above −7 Kcal/mol. Rutin again demonstrated the highest affinity (−12.861 Kcal/mol), followed by quercetin-3-O-galactoside (−10.243 Kcal/mol), and rosmarinic acid (−9.665 Kcal/mol) (Figure 8). In comparison, the co-crystallized inhibitor exhibited lower predicted docking score (−4.910 kcal/mol) than those of AcEE compounds (Table S1).

Against intracellular signaling proteins, the compounds exhibited moderate yet significant binding affinities, with seven compounds presenting affinities higher than −5 Kcal/mol. For Akt-1, luteolin showed the strongest affinity (−6.860 Kcal/mol), followed by rutin (−6.291 Kcal/mol), and 4-O-caffeoylquinic acid (−6.290 Kcal/mol), all exceeding the score of the reference inhibitor MK2206 (−3.940 kcal/mol), a known selective Akt-1 inhibitor (Table S1). Toward STAT3, quercetin-3-O-galactoside emerged as the top binder ligand (−7.269 Kcal/mol), followed by rutin (−6.738 Kcal/mol), whereas the co-crystallized inhibitor SI109 showed a predicted score of −6.740 kcal/mol (Table S1).

Moreover, docking against inflammatory cytokines further demonstrated appreciable ligand binding. For TNF-α, quercetin-3-O-galactoside showed the highest affinity (−7.657 Kcal/mol), followed by 4-O-caffeoylquinic acid (−7.501 Kcal/mol), and rutin (−6.795 Kcal/mol), all of which displayed higher docking scores than the co-crystallized ligand SPD304 (−3.330 kcal/mol) (Table S1). In the case of IL-1β, rosmarinic acid was found to be the strongest binder (−7.668 Kcal/mol), followed closely by rutin (−7.293 Kcal/mol). 

However, the reference inhibitor T9C showed a higher docking score of −8.470 kcal/mol (Table S1). Compared to other targets, AcEE constituents displayed weaker binding toward IL-6. The highest affinities were observed for rosmarinic acid (−5.570 Kcal/mol) and rutin (−5.163 Kcal/mol), while the remaining compounds showed binding energies below −4 Kcal/mol (Figure 8). In comparison, the synthetic small-molecule inhibitor LMT-28 exhibited a docking score of −4.150 kcal/mol (Table S1).

Analysis of protein–ligand interactions was carried out for representative complexes selected based on their binding affinity. For each hub protein, the ligand exhibiting the highest binding score was considered for detailed interaction analysis compared to the corresponding reference inhibitor and the selected complexes are provided in the supplementary data file. As depicted in Figure S10A, rutin was well accommodated within the kinase domain of the insulin receptor, forming a dense interaction network dominated by six hydrogen bonds involving Lys1057, Met1106, Arg1163, Asn1164, and Thr181. These interactions were complemented by multiple hydrophobic contacts with surrounding residues. The co-crystallized ligand established hydrogen bonds with Lys1057, Glu1104, and Met1106, together with a salt bridge involving Asp1110 and multiple hydrophobic contacts within the ATP-binding pocket (Figure S11A). A comparable interaction profile was observed for EGFR, in which rutin adopted a well-defined pose within the catalytic site and formed seven hydrogen bonds with key residues, including Ser720, Gln791, Met793, Pro794, and Asp800. These interactions were further supported by several hydrophobic contacts (Figure S10B). The EGRF co-crystallized inhibitor established key hydrogen bonds with Gln791 and Met793 within the catalytic pocket, together with multiple hydrophobic contacts, suggesting a comparable predicted binding mode (Figure S11B).

For Akt-1, the reference inhibitor MK2206 interacted primarily with Leu52 and Asn53 via hydrogen bonds, and with Tyr18 via pi-cation interaction (Figure S11C).

In comparison, in the luteolin-Akt-1 complex, luteolin was positioned within the pleckstrin homology (PH) domain, where it established five hydrogen bonds with Tyr18, Ile19, Arg23, Arg25, and Asn53, along with hydrophobic interactions with Phe55, Leu52, Phe27, and Trp22 (Figure S10C). Quercetine-3-O-galactoside demonstrated preferential binding with STAT3 and TNF-α. In complex with STAT3, quercetin-3-O-galactoside formed hydrogen bonds with Pro639, Glu638, Ser636, Tyr657, and Lys658, accompanied by hydrophobic contacts that supported its binding with the protein (Figure S10D). The co-crystallized inhibitor SI109 established several hydrogen bonds with Glu638, Tyr657, Ser636, Arg609, Glu594 (Figure S11D). In parallel, interaction analysis of the quercetin-3-O-galactoside-TNF-α complex revealed the formation of hydrogen bonds with Leu120, Gln61, Gln149, and Tyr151, together with surrounding hydrophobic contacts (Figure S10E). The co-crystallized inhibitor SPD304 interacted primarily with Gln149 and Tyr151 through hydrogen bonds, while engaging hydrophobic residues including Leu120, Tyr119, Leu57, Tyr59, and Ile155 (Figure S11E).

Finally, with the pro-inflammatory cytokines, IL-1β and IL-6, rosmarinic acid showed the most favorable interaction patterns. For IL-1β, the reference inhibitor T9C interacted with residues including Glu50, Lys93, Lys94, Lys97, and Asn102 within the binding pocket (Figure S11F). In contrast, rosmarinic acid adopted a distinct binding orientation, forming hydrogen bonds with Val3, Asn7, Lys63, and Leu67, together with surrounding hydrophobic contacts (Figure S10F). These results suggest an alternative predicted binding mode for rosmarinic acid within IL-1β.

Likewise, with IL-6, rosmarinic acid engaged with Lys171, Ar179, and Cys73 via hydrogen bonds, in addition to hydrophobic contacts and π-π interaction with Phe74 (Figure S10G). In contrast, the reference inhibitor LMT-28 interacted predominantly with Lys86 and Glu93, via hydrogen bonds together with residues lining the reported binding pocket (Figure S11G). This finding suggests a distinct predicted binding mode that warrants further experimental validation.

## 3. Discussion

To our knowledge, the present study provides the first comprehensive insight into the phytochemical composition and biological properties of *Astragalus cruciatus* Link, with a particular focus on its antidiabetic potential. Although species belonging to the genus *Astragalus* are widely recognized for their pharmacological importance, *A. cruciatus* Link has remained largely unexplored. In this context, the current findings establish a foundational understanding of its secondary metabolite profile and highlight this species as a promising natural source of therapeutically relevant bioactive compounds.

The extraction yield of Ac ethanolic extract (AcEE) was within the range reported for several *Astragalus* species obtained using alcoholic solvents, indicating a comparable recovery of soluble secondary metabolites. In our previous studies on the aerial parts of *A. gombo*, the aqueous extract and methanolic fraction showed relatively higher yields of 18.24% and 15.52%, respectively [27]. Similarly, studies on *Astragalus* species originating from Algeria and Turkey reported higher ethanolic yields than those obtained in the present work [30,33]. Such variations are commonly attributed to differences in solvent polarity, extraction conditions, plant part used, developmental stage, eco-geographical conditions, in addition to species-specific metabolic characteristics that influence the accumulation of secondary metabolites within the genus [34,35].

Phytochemical quantification further demonstrated that AcEE contains substantial levels of total phenolics and flavonoids, confirming the effectiveness of ethanol in extracting phenolic constituents from *A. cruciatus* Link. The phenolic and flavonoid contents obtained here were comparable to or higher than those reported for methanolic and ethanolic extracts of related Algerian taxa, including *A. gombo* and *A. armatus* [27,28], with *A. cruciatus* containing approximately two- to three-fold higher phenolic and flavonoid levels than *A. gombo*. Our findings also agree with those reported for aerial parts of *A. arpilobus* [29], highlighting *A. cruciatus* as a rich source of phenolic compounds within the genus. Such interspecific differences in polyphenol content are well documented and are largely influenced by both genetic determinants and environmental adaptation mechanisms shaping secondary metabolism.

In addition, Vorobyova and collaborators. [36] reported that extraction using mixtures of water and organic solvents can sometimes get a bit more yield.

LC-ESI-MS profiling revealed a chemical profile rich in phenolic acids and flavonoids. Notably, quinic acid was the most abundant phenolic acid in AcEE, with levels higher than those reported for methanolic and ethanolic extracts of the Algerian species *A. gombo* and *A. armatus* [27,28]. In contrast, the absence of quinic acid has been reported in methanolic whole-plant extracts and ethanolic leaf extracts of certain Turkish *Astragalus* species [33,37]. Notably, the predominance of hydroxycinnamic acids over hydroxybenzoic derivatives observed in our study is consistent with previous phytochemical studies reporting the abundance of hydroxycinnamic acids in *Astragalus* species [38,39].

Flavonoid profiling further revealed that flavonol glycosides constituted the predominant subclass in AcEE, with rutin identified as the major constituent, followed by cirsiliol and hyperoside. This profile is in agreement with numerous studies published between 2019 and 2025, which consistently demonstrated that flavonol glycosides represent the predominant class of compounds within the *Astragalus* genus [27,30,37,40,41]. More broadly, phytochemical investigations have identified over fifty flavonoids across *Astragalus* species, encompassing aglycones, iso-flavones, and their glycosides, with iso-flavonoid derivatives frequently representing dominant constituents [42].

The antioxidant activity of AcEE was assessed using complementary assays that evaluate different mechanisms, including electron-transfer capacity, radical scavenging, lipid peroxidation inhibition, and metal chelation. Overall, AcEE exhibited a broad antioxidant profile, consistent with its high phenolic and flavonoid contents. In the β-carotene-linoleic acid system, AcEE showed moderate inhibition of lipid peroxidation compared with BHA, in agreement with previous reports on related *Astragalus* extracts, where activity depends on the balance between hydrophilic and lipophilic antioxidants and their synergistic interactions [43,44,45]. Likewise, its metal-chelating activity was weaker than that of EDTA but comparable or superior to that reported for several *Astragalus* extracts [46]. However, the activity was lower than that observed for *A. armatus* and *A. gombo* [25,27], indicating interspecific variability in metal-binding phenolics. In contrast, in the ferric-reducing assay, AcEE showed good electron-donating capacity, exceeding values reported for methanolic extracts of *A. leporinus and A. ponticus* [46,47], and aqueous extracts from related Fabaceae taxa (*Genista saharae* and *Ononis angustissima*) [48,49]. Consistent with this, AcEE demonstrated notable DPPH radical-scavenging activity, comparable to several *Astragalus* species, with activity largely influenced by phenolic composition and solvent affinity [27,33,37]. This predominance of electron-transfer and radical scavenging mechanisms over lipid peroxidation is likely attributable to its high content of redox-active phenols, particularly flavonol glycosides and hydroxycinnamic acids.

The presence in AcEE of major antioxidant constituents such as quinic acid, rutin, hyperoside, protocatechuic acid, and p-coumaric acid provides a possible mechanistic basis for this activity, as these compounds are recognized hydrogen- or electron-donating antioxidants capable of stabilizing DPPH radicals [28,50]. Moreover, quinic acid and its derivatives have previously been identified as effective DPPH scavengers [51], supporting the contribution of the abundant quinic acid detected in *A. cruciatus* Link, although the individual contribution of each compound remains to be experimentally established.

Following the phytochemical characterization and antioxidant evaluation, the antidiabetic potential of AcEE was investigated through in vitro inhibition of α-amylase and α-glucosidase, two key enzymes involved in dietary carbohydrate digestion and established therapeutic targets for controlling postprandial hyperglycemia in type 2 diabetes mellitus [52]. Clinically available inhibitors, including acarbose, voglibose, and miglitol, effectively reduce postprandial glucose excursions, but their long-term use is frequently limited by gastrointestinal adverse effects such as abdominal discomfort, bloating, and diarrhea [53,54]. Consequently, this situation has intensified the interest in plant-derived enzyme inhibitors as safer alternatives or complementary therapeutic agents. In this context, AcEE exhibited significant in vitro inhibitory activity against both α-amylase and α-glucosidase, suggesting that *A. cruciatus* may influence postprandial glucose metabolism while potentially minimizing adverse effects associated with conventional therapies.

The observed enzyme inhibition can be attributed to the rich phenolic and flavonoid composition of the extract. In fact, phenolic acids and flavonoids are known to inhibit α-amylase and α-glucosidase due to their characteristic structures rich in hydroxyl groups, which in turn support their binding to enzymes’ active sites, thereby altering their catalytic activity [55]. In line with this, rutin is already well known to exert considerable anti-diabetic and anti-hyperglycemic effects through multiple mechanisms, including its capacity to reduce glucose absorption from the small intestine via inhibition of α-amylase and α-glucosidase [56]. Similarly, quercetin-3-O-galactoside, kaempferol, apigenin, and quinic acid, among others, have been reported to exhibit significant antidiabetic activity and to effectively suppress both α-amylase and α-glucosidase activities [57,58,59]. However, because the present study evaluated the crude extract rather than isolated constituents, the contribution of individual compounds cannot be established. The observed activity is therefore more likely to result from the combined effects of multiple constituents within the extract, a hypothesis that requires further experimental validation.

The MTT assay was performed as a preliminary screening method to evaluate the effects of AcEE on cellular viability and provide an initial assessment of its in vitro cytotoxicity. Owing to its simplicity, reproducibility, and sensitivity, the MTT assay is widely used to screen the cytotoxic potential of plant extracts prior to more comprehensive toxicological investigations [60,61]. In the present study, AcEE exhibited IC_50_ values of 403.40 ± 23.26 μg/mL in AML12 cells and 396.30 ± 44.33 μg/mL in HepG2 cells. According to the National Cancer Institute (NCI) screening criteria, crude plant extracts with IC_50_ values below 20–30 μg/mL are generally considered cytotoxic, whereas extracts with substantially higher IC_50_ values are regarded as exhibiting low cytotoxicity [60,61,62]. Accordingly, the IC_50_ values obtained for AcEE indicate low cytotoxicity under the present experimental conditions.

Molecular docking studies were subsequently conducted to predict the interactions of the identified phytochemicals with α-amylase and α-glucosidase. Overall, flavonoids, particularly glycosylated derivatives, displayed more favorable predicted docking scores than simple phenolic acids. Among them, rutin exhibited the highest predicted docking scores for both enzymes. This trend may be explained by their larger molecular scaffolds and functional group richness. The abundance of hydroxyl groups and extended aromatic rings in flavonoids facilitates extensive hydrogen bonding and π-π interactions within enzyme active sites, thereby stabilizing ligand-protein complexes, whereas phenolic acids, despite their bioactivity, possess simpler scaffolds that may limit molecular interactions and result in comparatively weaker binding.

The favorable binding interactions predicted for rutin in the present study are supported by previous experimental investigations demonstrating its inhibitory activity against both α-amylase and α-glucosidase. Collectively, these studies have reported α-amylase IC_50_ values ranging from 22.0 μg/mL to 6.73 mg/mL and α-glucosidase IC_50_ values ranging from 7.86 μg/mL to 1.30 mg/mL. Specifically, Dubey and collaborators [63] demonstrated concentration-dependent inhibition of both α-amylase and α-glucosidase, with rutin producing 53.66% and 52.56% inhibition, respectively, at 250 μg/mL. Maradesha and collaborators. [64] observed markedly stronger inhibition, reporting IC_50_ values of 22.0 μg/mL and 7.86 μg/mL against α-amylase and α-glucosidase, respectively. In contrast, Zhang and coillaborators. [65] reported comparatively weaker α-amylase inhibition (IC_50_ = 6.730 mg/mL) while confirming moderate α-glucosidase inhibition (IC_50_ = 0.1895 mg/mL). Although the reported inhibitory potencies differ considerably, these independent studies consistently examine rutin as an inhibitor of both carbohydrate-hydrolyzing enzymes, lending biological support to the favorable binding interactions predicted by the present molecular docking analyses.

MM/GBSA calculations further strengthened the docking predictions by supporting the energetic stability of the rutin-enzyme complexes. Unlike docking scores, which primarily estimate the quality of ligand fitting within the binding pocket, MM/GBSA provides a more comprehensive estimation of binding free energy by incorporating molecular mechanics and solvation energy terms. Furthermore, induced fit docking and molecular dynamics simulations revealed that rutin maintains a stable binding conformation within the active sites of both enzymes, further supporting the stability of protein–ligand complexes. These complementary approaches increase confidence in the predicted binding modes but should not be interpreted as experimental evidence of binding affinity or inhibitory potency. Consequently, the computational analyses provide mechanistic hypotheses that complement in vitro enzyme inhibition assays and warrant further validation using purified compounds and enzyme-based biochemical studies. Moreover, our results suggest rutin as a computationally predicted candidate with favorable binding energetics. Nevertheless, given its low mass fraction in the extract, its direct contribution to the observed in vitro enzyme inhibition is likely limited. Instead, the observed activity is more probably attributable to synergistic interactions among multiple bioactive constituents.

While the phytochemical characterization and in vitro assays demonstrated the antioxidant and enzyme inhibitory activities of AcEE, they do not fully capture the complexity of molecular interactions underlying T2DM. Therefore, network pharmacology was employed as a complementary computational approach to identify potential molecular targets and pathways associated with the observed bioactivities. Protein–protein interaction (PPI) analysis identified seven hub proteins as central regulatory nodes, namely interleukin-6 (IL-6), tumor necrosis factor-alpha (TNF-α), interleukin-1 beta (IL-1β), protein kinase B (Akt-1), signal transductor and activator of transcription 3 (STAT3), epidermal growth factor receptor (EGFR), and insulin receptor (INSR), all of which have established roles in insulin signaling, glucose homeostasis, inflammation, and oxidative stress.

Among these hubs, INSR and Akt-1 form the core of the insulin-signaling pathway. The INSR/PI3K/AKT pathway is essential for maintaining glucose homeostasis by regulating glucose uptake, glycogen synthesis, and hepatic glucose production, and its dysregulation is a hallmark of insulin resistance and T2DM [66,67,68]. Downstream of INSR, protein kinase B (Akt-1) functions as a master regulator of insulin-mediated metabolic responses. Akt occupies a critical position within the insulin signaling cascade. Upon PI3K-dependent activation, Akt promotes glucose uptake through GLUT4 translocation in muscle and adipose tissue, suppresses hepatic gluconeogenesis via inhibition of the FoxO1 transcription factor, and enhances glycogen synthesis through inhibition of glycogen synthase kinase-3β (GSK3β) [69,70]. Impaired AKT signaling is a hallmark of insulin resistance and contributes to defective glucose homeostasis in T2DM [71,72]. From a therapeutic perspective, strategies aimed at restoring INSR/PI3K/Akt signaling have consistently demonstrated beneficial metabolic effects in experimental models [73,74,75]. The identification of INSR and AKT1 as hub proteins therefore suggests that AcEE constituents may influence insulin signaling pathways. However, these interactions are computational predictions and require experimental validation before mechanistic conclusions can be drawn.

On the other hand, the identification of inflammatory mediators within the PPI network supports the recognized contribution of chronic low-grade inflammation to T2DM pathogenesis [76,77]. This concept emerged from landmark studies in the early 1990s, notably the work of Hotamisligil and colleagues, who demonstrated elevated expression of TNF-α in the adipose tissue of obese rodents and showed that neutralization of TNF-α signaling improved insulin sensitivity [7,78].

Among the inflammatory hubs identified, TNF-α is among the most studied cytokines linking obesity, inflammation, and insulin resistance. Experimental studies have demonstrated that TNF-α directly impairs insulin signaling through activation of NF-κB and JNK pathways, leading to reduced insulin efficiency [79,80]. IL-6, another central hub in our PPI network, serves as both a marker and a mediator of low-grade inflammation in T2DM. Elevated IL-6 levels are consistently observed in patients with T2DM and are strongly associated with increased risk of disease development and cardiovascular complications. IL-6 signals mainly via the JAK/STAT pathway, with STAT3 acting as a key downstream transcriptional regulator. Persistent IL-6-STAT3 signaling contributes to inflammatory gene expression, endothelial dysfunction, and impaired insulin responsiveness [80,81].

For epidermal growth factor receptor (EGFR), although its association with T2DM has not been definitively defined as causal, increasing evidence supports its involvement in both metabolic dysregulation and diabetes-related complications [82]. Crosstalk between EGFR and insulin signaling pathways, particularly through PI3K/AKT, may influence insulin sensitivity [82]. At the same time, aberrant EGFR activation has been reported in adipose tissue from obese individuals at high risk of developing T2DM [83].

Importantly, EGFR signaling has been implicated in the development of T2DM-related complications, specifically microvascular dysfunction. In which its disruption contributes to damage to small blood vessels, affecting organs such as the kidneys and glomerular and tubular cells. EGFR was also involved in vascular dysfunction, contributing to hypertension and atherosclerosis observed in diabetic patients [84,85]. From a therapeutic perspective, inhibition of EFGR has shown promising effects on both metabolic control and diabetic complications. Studies have demonstrated that EGFR inhibitors, including imatinib, erlotinib, and sunitinib, improve insulin sensitivity and glucose tolerance [82,86,87].

Pathway enrichment analysis was subsequently performed to contextualize these targets within the biological processes associated with T2DM. The enriched pathways revealed a strong overlap between metabolic, chronic inflammation, oxidative stress, and vascular injury pathways, all of which are central to T2DM pathogenesis and its complications. The most significantly enriched pathway was AGE-RAGE signaling, suggesting the interference of ACEE constituents to prevent hyperglycemia-driven molecular damage during disease progression. It is well established that in diabetic conditions, due to chronic hyperglycemia, circulating and tissue levels of advanced glycation end products (AGEs) are markedly elevated and are closely associated with both disease progression and the onset of diabetic complications. The interaction of AGEs with their receptor RAGE triggers sustained activation of the AGE-RAGE signaling axis, leading to transcriptional activation that promotes chronic inflammation, oxidative stress, insulin resistance, and β-cell dysfunction. Persistence of this damaging cascade disrupts normal cellular homeostasis, and it is recognized as a major contributor to the development of cardiovascular, renal, and neuronal complications [88,89,90].

Consistent with the identified inflammatory hub genes, pro-inflammatory pathways, including IL-17 and TNF-α pathways, were significantly enriched. These pathways are already known as key drivers of cytokine production and stress kinase activation, processes known to sustain chronic low-grade inflammation and disrupt insulin signaling. Enrichment of the insulin resistance pathway further supports the central role of AcEE constituents in modulating insulin signaling. Additionally, pathways linked to lipid metabolism and vascular dysfunction, notably lipid and atherosclerosis, were predominantly represented, underscoring the mechanistic interplay between dyslipidemia, endothelial injury, and inflammation that underlies the elevated cardiovascular risk associated with T2DM pathology.

The molecular docking analysis provided additional mechanistic support for the network pharmacology findings by demonstrating favorable interactions between AcEE constituents and key targets implicated in T2DM. Among all tested ligands, rutin exhibited strong predicted binding affinity toward INSR, suggesting its possible interaction with this protein. While our docking analysis indicates favorable binding of rutin to INSR, functional validation is required. Notably, this predicted interaction is consistent with independent experimental reports demonstrating that rutin enhances insulin receptor kinase activity, promotes IRS/PI3K/Akt signaling, and facilitates GLUT4 translocation, thereby improving glucose uptake [91,92]. In type 2 diabetic models, rutin treatment restored glucose homeostasis by activating the IRS/PI3K/Akt/GSK-3β pathway, supporting its role in improving insulin responsiveness [93]. Rutin also demonstrated strong binding affinity toward EGFR, suggesting a potential for multitarget engagement; however, whether this interaction results in inhibition or activation requires experimental elucidation.

For Akt-1, luteolin exhibited the highest predicted binding affinity, which aligns with literature reports of its antidiabetic activity, though our docking data alone do not establish functional modulation. Experimental studies have shown that luteolin enhances glucose consumption and glycogen synthesis in insulin-resistant HepG2 cells by activating the PI3K/Akt cascade, while suppressing oxidative stress and AGE formation [94,95]. Regarding inflammatory regulators, our findings showed that quercetin-3-O-galactoside, also known as hyperoside, exhibited the highest binding affinities toward STAT3 and TNF-α. This computational finding is supported by literature indicating that hyperoside can suppress TNF-α production in LPS-induced proliferation and migration of inflammatory cells [96,97]. Moreover, hyperoside has been reported to exert its effects through the modulation of the STAT/Akt/ERK signaling pathway [98]. Both in vivo and in vitro studies have demonstrated that hyperoside increases phosphorylation of JAK2 and STAT3, highlighting its capacity to regulate inflammatory signaling [99]. For IL-1β, rosmarinic acid displayed the strongest binding affinity, in agreement with the previous literature documenting its anti-inflammatory activity. Rosmarinic acid has been shown to inhibit the production of pro-inflammatory mediators and suppress key inflammatory pathways [100].

Collectively, the docking results reinforce the systems-level findings by demonstrating that AcEE constituents engage both metabolic targets and inflammatory regulators with high affinity. This multitarget interaction profile supports a coordinated mechanism through which AcEE may simultaneously enhance insulin signaling and suppress chronic inflammation, a dual action that is particularly relevant to the complex immune-metabolic pathology of T2DM.

### Limitations

Despite the promising findings, the present study presents some limitations. First, the enzyme inhibition assay evaluations were conducted in vitro, which cannot fully capture the complexity of in vivo glucose metabolism, insulin sensitivity, and tissue-specific pharmacodynamics. Although our study identified rutin as the most abundant flavonoid in AcEE, its mass fraction is insufficient to account for the observed enzyme inhibition at the extract’s IC_50_. The present study did not determine the IC_50_ of pure rutin under identical experimental conditions, nor did it assess the inhibitory activity of other major constituents individually. Therefore, the attribution of activity to specific compounds remains speculative. Future studies should include dose–response assays of pure compounds to evaluate potential additive or synergistic effects among the polyphenolic constituents. Also, the absence of intestinal cell lines (e.g., Caco-2 or HT-29) is notable, considering that α-amylase and α-glucosidase inhibition occurs at the intestinal level. Second, although molecular docking and molecular dynamics simulations provide mechanistic insight into ligand-protein interactions, these approaches are predictive and require experimental validation. Finally, there is a lack of mechanistic cellular validation, such as Western blotting or qPCR, to confirm modulation of the PI3K/Akt, AGE-RAGE, or inflammatory pathways predicted by network pharmacology.

## 4. Materials and Methods

### 4.1. Chemicals and Reagents

All the chemicals and solvents were of analytical quality. Chemical standards with a purity exceeding 98% were purchased from Sigma Chemical Co. (St. Louis, MO, USA). Butylated hydroxyanisole (BHA); ferrozine; 1,1-diphenyl-2-picrylhydrazyl (DPPH); linoleic acid; along with additional substances such as potassium ferricyanide, trichloroacetic acid (TCA), ferric dichloride (FeCl_2_), ethylenediaminetetraacetic acid (EDTA), sodium hydroxide, and enzymes, as well as alpha-amylase and alpha-glucosidase, and the HepG2 cell line sourced from Sigma Chemical Co. (St. Louis, MO, USA). The AML12 cell line was obtained from the American Type Culture Collection (CRL-2254, ATCC, Manassas, VA, USA).

### 4.2. Plant Collection and Identification

Plant material was collected in May 2019, from Lichana (Tolga region), Algeria (136 m of altitude, 34°43′0″ N/5°25′60″ E). The species was taxonomically identified at the Technical Institute for The Development of Saharan Agronomy (ITDAS) as *Astragalus cruciatus* Link. A voucher specimen (FA-029) was deposited in ITDAS herbarium of Biskra, Algeria. All the organs (leaves, flowers, and stems) of the aerial parts were separated, dried in the shade at room temperature under protection from humidity, and subsequently ground into a fine powder, which was used to carry out the various analyses.

### 4.3. Extraction Procedure

The phenolic compounds of *A. cruciatus* were extracted by maceration using a hydro-ethanolic solvent system. Briefly, 50 grams (g) of powdered plant material were mixed with 500 mL of an ethanol/distilled water solution (80:20, *v*/*v*) and macerated at 25 °C for 72 h. The extract was then filtered through a 0.45 µm Whatman filter and centrifuged at 4000 rpm for 15 min. The supernatant was collected and evaporated to dryness under reduced pressure at 50 °C. The concentrated extract was subsequently lyophilised, weighed to determine extraction yield, and stored at 4 °C until further analysis.

### 4.4. Colorimetric Assays

#### 4.4.1. Determination of Total Phenolic Content (TPC)

Total phenolic content (TPC) was quantified using the Folin–Ciocalteu colorimetric method, as described by [101]. Briefly, 200 µL of the extract were mixed with 1 mL of Folin–Ciocalteu reagent (diluted 1:10 with distilled water) and 0.8 mL of sodium carbonate solution (7.5%, *w*/*v*). The reaction mixture was vortexed and incubated at room temperature for 30 min. Absorbance was measured at 765 nm using a spectrophotometer (Jenway/Bibby, model 6310 visible screening, Scientific Ltd, Chelmsford, UK). TPC was calculated from a gallic acid calibration curve, prepared under the same conditions, and expressed as milligrams of gallic acid equivalents per gram of dry weight (mg GAE. g^−1^ DW). All measurements were performed in triplicate.

#### 4.4.2. Determination of Total Flavonoid Content (TFC)

Total flavonoid content (TFC) was determined using the aluminum chloride colorimetric assay following [102]. An aliquot of diluted extract was mixed with 75 µL of sodium nitrate solution and incubated for 6 min. Subsequently, 0.15 mL of aluminum chloride solution (100 g/L) was added. Following a 5 min incubation, 0.5 mL of sodium hydroxide solution was introduced. Then, the final volume was adjusted to 2.5 mL with distilled water and thoroughly vortexed to ensure homogeneity. Absorbance was recorded at 510 nm against a reagent blank. TFC was calculated from a quercetin calibration curve and expressed as milligrams of quercetin per gram of dry weight (mg QE. g^−1^ DW). All samples were analyzed in triplicate.

### 4.5. Liquid Chromatography-Electrospray Ionization-Tandem Mass Spectrometry (LC-ESI-MS) Analysis

The dried ethanolic extract was re-solubilized in its respective solvent, and the obtained solution (4 mg/mL) was filtered through a 0.45 μm membrane before chromatographic injection. LC-ESI-MS analyses were performed on an LCMS-2020 quadrupole mass spectrometer (Shimadzu, Kyoto, Japan) equipped with an electrospray ionization source operating in negative ion mode. The mass spectrometer was connected in line to an ultra-fast liquid chromatography system comprising an LC-20AD XR binary pump, an SIL-20AC XR auto-sampler, a CTO-20AC column oven, and a DGU-20A 3R degasser (Shimadzu, Kyoto, Japan). An Aquasil C18 column (Thermo Electron, Dreieich, Germany) (150 mm × 3 mm, 3 μm) preceded by an Aquasil C18 guard column (10 mm × 3 mm, 3 μm, Thermo Electron) was applied for analysis. The mobile phase was composed of A (0.1% formic acid in H_2_O, *v*/*v*) and B (0.1% formic acid in methanol, *v*/*v*) with a linear gradient elution: 0–45 min, 10–100% B; 45–55 min, 100% B. Re-equilibration duration was 5 min between individual runs. The flow rate of the mobile phase was 0.4 mL/min, the column temperature was maintained at 40 °C and the injection volume was 5 μL. Spectra were monitored in SIM (Selected Ion Monitoring) mode and processed using Shimadzu LabSolutions LC-MS software. High-purity nitrogen was used as the nebulizer and auxiliary gas. The mass spectrometer was operated in negative ion mode with a capillary voltage of −3.5 V, a nebulizing gas flow of 1.5 L/min, a dry gas flow rate of 12 L/min, a DL (dissolving line) temperature of 250 °C, a block source temperature of 400 °C, a voltage detector of 1.2 V, and the full scan spectra from 50 to 2000 Da.

### 4.6. Antioxidant Activity

#### 4.6.1. β-Carotene/Linoleic Acid Bleaching Test

The ability of AcEE to inhibit β-carotene bleaching was evaluated according to the method of [103]. A β-carotene/linoleic acid stock solution was prepared by dissolving 0.5 mg of β-carotene, 25 μL of linoleic acid, and 200 μL of Tween 40 in 1 mL of chloroform. The chloroform was then completely evaporated under reduced pressure using a rotary evaporator at 40 °C. Subsequently, 100 mL of bi-distilled water was added, and the mixture was vigorously emulsified. The resulting emulsion was freshly prepared prior to each experiment. Aliquots (2.5 mL) of the emulsion were transferred into test tubes containing 0.5 mL of the extract at different concentrations. The mixtures were incubated at 50 °C for 2 h, after which the absorbance was measured at 470 nm. Butylated hydroxyanisole (BHA) was used as a standard antioxidant, while the control sample contained no extract. The antioxidant activity was expressed as the percentage inhibition of β-carotene bleaching and calculated as follows:

β-carotene bleaching inhibition (%) = [1 − (A_0_ − A_t_)/(A′_0_ − A′ₜ)] × 100

where A_0_ and A_t_ represent the absorbance of the sample at time zero and after 2 h, respectively, and A′_0_ and A′ₜ correspond to the absorbance of the control at time zero and after 2 h, respectively.

#### 4.6.2. Ferrous-Ion Chelating (FIC) Activity

The ferrous ion chelating activity of AcEE was evaluated according to the method described by [104]. Briefly, 100 μL of AcEE at various concentrations was mixed with 50 μL of FeCl_2_·4H_2_O solution (2 mM) and incubated at room temperature for 5 min. The reaction was initiated by the addition of 100 μL of ferrozine solution (5 mM), followed by addition of 2750 μL of deionized water. The mixture was vigorously shaken and allowed to stand at room temperature for 10 min. Subsequently, the absorbance was measured at 562 nm. The chelating activity was expressed as the percentage inhibition of the ferrozine–Fe^2+^ complex formation and calculated using the following equation:

Ferrous ion-chelating activity (%) = [(A_control − A_sample)/A_control] × 100

where A_control is the absorbance of the control reaction (without extract), A_blank is the absorbance of the blank (without ferrozine), and A sample is the absorbance of the reaction mixture containing the extract. EDTA was used as the positive control.

#### 4.6.3. DPPH Radical Scavenging Assay

The free radical scavenging activity of AcEE was evaluated using the DPPH assay according to the method of [105], with minor modifications. Briefly, 500 µL of the extract at various concentrations were mixed with 375 µL of ethanol and 125 µL of a 0.2 mM DPPH solution (prepared in ethanol). The reaction mixtures were incubated in the dark at room temperature for 60 min. The absorbance was measured at 517 nm using a spectrophotometer (Jenway/Bibby, model 6310 visible screening, Scientific Ltd, Chelmsford, UK). The percentage of DPPH radical scavenging activity was calculated using the following equation:

Scavenging activity (%) = [(A_control − A_sample)/A_control] × 100

where A_control represents the absorbance of the control reaction containing all reagents except the extract, and *A_sample* represents the absorbance in presence of the extract/standard. The blank consisted of all reagents except the DPPH solution. Butylated hydroxyanisole (BHA) was used as a positive control. The IC_50_ value, defined as the concentration required to scavenge 50% of DPPH radicals, was determined from the plot of inhibition percentages against extract concentrations.

#### 4.6.4. Ferric Reducing Power Assay (FRAP)

The reducing capacity of AcEE was evaluated according to the method of [106]. Briefly, 0.5 mL of AcEE at various concentrations was mixed with 1.25 mL of phosphate buffer (0.2 M, pH 6.6) and 1.25 mL of potassium ferricyanide solution (1%, *w*/*v*). The mixture was incubated at 50 °C for 30 min. Then, 1.25 mL of trichloroacetic acid (10%, *w*/*v*) was added, and the mixture was centrifuged at 2500× *g* for 10 min. An aliquot (1.25 mL) of the supernatant was then mixed with 1.25 mL of distilled water and 0.25 mL of ferric chloride solution (0.1%, *w*/*v*). After 10 min of reaction, the absorbance was measured at 700 nm. The IC_50_ value (µg/mL), defined as the concentration required to achieve an absorbance of 0.5, was calculated by linear regression analysis. Butylated hydroxyanisole (BHA) was used as the reference antioxidant.

#### 4.6.5. Total Antioxidant Activity (TAC)

The total antioxidant capacity of AcEE was determined using the phosphomolybdate method as described by [107]. Briefly, 0.3 mL of the extract was mixed with 3 mL of the reagent solution containing sulfuric acid (0.6 M), sodium phosphate (28 mM), and ammonium molybdate (4 mM). The reaction mixtures were capped and incubated at 95 °C for 90 min. After cooling to room temperature, the absorbance was measured at 695 nm against a reagent blank. Total antioxidant capacity was expressed as milligrams of gallic acid equivalents per gram of dry weight (mg GAE/g DW).

### 4.7. In Vitro Antidiabetic Activity

#### 4.7.1. Alpha-Amylase Inhibition Assay

The inhibitory potential of Ac ethanolic extract was evaluated using a spectrophotometric technique [108], with slight modifications. The extract was tested at the concentrations of 10, 100, 200, 400, and 800 μg/mL. The reaction solution comprised 200 µL of sample and 200 µL of α-amylase solution; the mixture was pre-incubated for 10 min at 37 °C. Subsequently, the reaction was initiated by adding 200 μL of starch (0.1%), followed by incubation at 37 °C for 15 min. The reaction was then stopped by the addition of 25 μL of 3,5-dinitro salicylic acid. A blank solution was prepared by substituting the extract with 200 μL of buffer solution. Acarbose was used as a positive control and the absorbance of the sample and blank was recorded at 540 nm. α-Amylase inhibitory activity was determined using the following formula:

% Inhibition = [control absorbance − sample absorbance)/control absorbance] × 100

#### 4.7.2. Alpha-Glucosidase Inhibition Assay

The α-glucosidase inhibitory activity of AcEE was evaluated using a spectrophotometric method, following the protocol of [109], with slight modifications. Briefly, 200 μL of the extract at various concentrations (10, 100, 200, 400, and 800 μg/mL) were mixed with 50 μL of α-glucosidase solution in phosphate buffer (100 mM, pH 6.8) and pre-incubated at 37 °C for 15 min. The enzymatic reaction was initiated by adding 50 μL of p-nitrophenyl-α-D-glucopyranoside (pNPG) and incubating the mixture at 37 °C for 20 min. The reaction was terminated by addition of 50 μL of sodium hydroxide solution (0.2 M). Absorbance readings were taken at 405 nm, and the α-glucosidase inhibitory activity was calculated using the following formula:

% Inhibition = [(X_A_ − X_B_)/X_A_] × 100

where X_A_ represents the absorbance of the control reaction (without the extract), and X_B_ is the absorbance in the presence of the extract or the reference inhibitor (acarbose).

### 4.8. Cytotoxicity Assay

The cytotoxic activity of AcEE was evaluated using the MTT test, according to the method of [110], with slight modifications. The human hepatocellular carcinoma cell line (HepG2) and the normal mouse liver cell line (AML12) were obtained from the American Type Culture Collection (ATCC, USA). Cells were cultured in DMEM medium supplemented with 10% fetal bovine serum (FBS), 2 mM L-glutamine, 1 mM sodium pyruvate, 100 U/mL penicillin, 100 μg/mL streptomycin, and 250 μg/mL fungizone. Then, the specimens were maintained in a humidified incubator at standard conditions of 37 °C and 5% CO_2_. For the assay, cells were treated with AcEE at concentrations ranging from 10 to 800 μg/mL and incubated at 37 °C for 72 h. For the control group, cells were treated with a concentration of ethanol (vehicle-control cells) that matched the concentration of ethanol present in the extract-treated cells. Then, cells were washed with PBS solution and incubated with 10 μL of MTT solution (at a concentration of 5 mg/mL) for 4 h. The absorbance of the reduced MTT was recorded at 570 nm. The 50% inhibitory concentrations (IC_50_) were determined from dose–response curves using GraphPad Prism software (v8.4.0, San Diego, CA, USA). HepG2 and AML12 were treated for 72 h with increasing doses of the positive control cisplatin, and compared to the DMSO-treated vehicle-control cells. IC_50_ values of cisplatin in A549 cells were measured at the three time points.

### 4.9. In Silico Studies

#### 4.9.1. Molecular Docking

Molecular modeling was employed to investigate the binding affinities and interaction modes of AcEE’s constituents within the catalytic sites of α-amylase and α-glucosidase. The 3D structures of the compounds, along with the reference inhibitor acarbose, were obtained from the PubChem database in SDF format and processed using Schrödinger’s LigPrep module (Schrödinger release 2021, LLC, New York, NY, USA), where protonation and tautomeric states were generated at physiological pH (7.0 ± 2.0), and geometries were minimized with the OPLS4 force field. In parallel, crystal structures of α-amylase (PDB ID: 1OSE) and α-glucosidase (PDB ID: 3A4A) were retrieved from the Protein Data Bank and refined using Schrödinger’s Protein Preparation Wizard (Schrödinger release 2021, LLC, New York, NY, USA). This step ensured structural integrity through correction of bond orders, optimization of hydrogen bonding networks, adjustment of protonation states, and energy minimization. Receptor grids were then centered on the catalytic pockets, defined by co-crystallized inhibitors and key active-site residues. Finally, docking simulations were executed using the Glide program (Schrödinger release 2021, LLC, New York, NY, USA) in extra precision (XP) mode, enabling reliable prediction of binding poses and scoring [111].

#### 4.9.2. MMGBSA Binding Free Energy Calculation

The binding free energies (ΔG_bind) of the docked protein–ligand complexes were estimated using the Molecular Mechanics/Generalized Born Surface Area (MM/GBSA) method implemented in the Prime module of the Schrödinger suite (Schrödinger 2021, LLC, New York, NY, USA). The protein–ligand complexes obtained from Glide XP docking were used as input structures. The OPLS4 force field was employed for energy calculations, and the VSGB implicit solvent model was applied to account for solvation effects [111].

#### 4.9.3. Induced Fit Docking (IFD)

In this study, we performed induced-fit docking (IFD) on the top-ranked compound, rutin, to predict the effect of flexible ligand docking on protein structure, moving beyond rigid receptor docking. The IFD option in Mastro (Schrödinger release 2021, LLC, New York, NY, USA) was used. After preparing the target, a standard protocol was employed to generate an enclosing box around the centroid of the ligands to be docked, using the native ligands from 1OSE and the A3A4 protein receptors. Then, an initial Glide docking for each compound was carried out. The van der Waals radii values were set to 0.7 for the receptor and 0.5 for the ligands. Residues within 5.0 Å of the binding pocket were refined using Prime, followed by side chain minimization. Final docked poses were re-docked against the refined protein using default Glide settings [110].

#### 4.9.4. Molecular Dynamics (MD) Simulations

Following docking, the top-scoring complexes were subjected to molecular dynamics simulations using the Desmond package (Version 2021, Schrödinger, LLC, New York, NY, USA) to evaluate their conformational stability and the persistence of intermolecular interactions over time. Simulated systems were properly prepared prior to calculations, in which each complex was placed in an orthorhombic simulation box and solvated using the SPC water model, with Na^+^ and Cl^−^ ions added at physiological concentrations to neutralize the system. System parametrization was performed using the OPLS4 force field, applied to both proteins and ligands. Energy minimization was carried out for 2000 steps using the steepest descent method to remove steric clashes, followed by equilibration under the isothermal-isobaric (NPT) ensemble at 300 K and 1 bar. Subsequently, production simulations were conducted for 100 ns, and trajectories were recorded at regular intervals for structural and interaction analyses [112].

### 4.10. Network Pharmacology

#### 4.10.1. Compounds Standardization and Targets Mapping

Chemical constituents identified in the AcEE were subjected to target mapping using a multi-database strategy. Canonical SMILES retrieved from the PubChem database were used as molecular descriptors for downstream analyses. Subsequently, the putative molecular targets corresponding to each compound were predicted from SwissTargetPrediction, STITCH, SuperPred (accessed on 4 January 2026), and the Comparative Toxicogenomics Database (CTD, accessed on 10 January 2026) databases [113,114]. To ensure clinical relevance, target prediction was restricted to *Homo sapiens*. The remaining targets were unified, with gene nomenclature standardized using the UniProtKB database, and redundant entries were removed to obtain a non-redundant compound-associated target set.

#### 4.10.2. Acquisition of Disease-Related Molecular Targets

Molecular targets implicated in type 2 Diabetes Mellitus (T2DM) were collected from GeneCards and OMIM databases (accessed on 3 January 2026) using ‘Type 2 Diabetes Mellitus’ as the search keyword [115]. Following data integration, duplicates were removed to yield a unified target dataset. The intersection between compound-associated targets and disease-related targets was subsequently identified through Venn diagram analysis using the jvenn online tool (https://jvenn.toulouse.inra.fr/app/index.html, accessed on 15 January 2026). The overlapping targets were considered key molecular nodes through which AcEE constituents may collectively modulate T2DM-related biological processes at a systems level.

#### 4.10.3. Protein–Protein Interaction (PPI) Network Construction and Topological Analysis

The protein–protein interaction (PPI) network was constructed using the STRING database (v. 12.0; https://string-db.org/, accessed on 12 January 2026). The analysis was restricted to *Homo sapiens*, and only interactions supported by high-confidence evidence were retained by applying a minimum interaction score threshold of 0.7, with a false discovery rate (FDR) set at 1%. Proteins lacking interaction links under these criteria were excluded. The resulting network was imported into Cytoscape software (version 3.10.2) for topological characterization [116]. Network topology was quantitatively evaluated using multiple centrality parameters to assess the relative importance of individual nodes within the interaction network. CytoHubba plugin-based algorithms, namely degree, closeness centrality, betweenness centrality, maximal clique centrality (MNC), and edge percolated component (EPC), were applied to identify the top 10 highly influential hub genes [117,118]. Proteins exhibiting consistently high rankings across these topological measures were considered central regulatory nodes within the PPI network.

#### 4.10.4. Gene Ontology (GO) and KEGG Pathways Enrichment Analysis

Gene Ontology (GO) and Kyoto Encyclopedia of Genes and Genomes (KEGG) pathway enrichment analyses were performed using the ShinyGO (version 0.85.1) online tool [119]. GO analysis annotated the overlapping target set across the biological process (BP), molecular function (MF), and cellular component (CC) categories, while KEGG analysis identified significantly enriched signaling pathways. Enrichment results were filtered using a false discovery rate (FDR) threshold of 0.01. The top 20 enriched GO terms and KEGG pathways, ranked by statistical significance, were selected for subsequent analysis and visualization.

#### 4.10.5. Molecular Docking Analysis

Molecular docking was performed on hub targets identified from the PPI network to structurally support the network pharmacology analysis. Hub proteins selected based on topological parameters were used as receptor models for docking simulations.

The docking protocol was validated by redocking the co-crystallized ligand of each protein into its binding site. When no suitable co-crystallized small-molecule ligand was available, a reported reference inhibitor was used for comparative analysis. The compounds were subsequently docked under identical conditions described in the molecular docking section above, and their predicted docking scores and binding interactions were compared with those of the corresponding reference ligands.

### 4.11. Statistical Analysis

Statistical analysis was carried out using GraphPad Prism v. 8.4.0. Experimental procedures were performed in triplicate (*n* = 3), and the results were presented as the mean ± standard deviation (SD). One-way analysis of variance (ANOVA) was performed, followed by Dunnett’s post hoc test, with a *p*-value of less than 0.05 considered statistically significant.

## 5. Conclusions

This study systematically investigated the antidiabetic potential of *Astragalus cruciatus* Link and provided preliminary insights into the molecular mechanisms underlying its bioactivity through complementary phytochemical, in vitro, and computational approaches. The investigated ethanolic extract was characterized by a phenolic and flavonoid-rich profile, with rutin and quinic acid identified as the major compounds. The extract exhibited marked antioxidant activity across multiple in vitro assays, suggesting that its phenolic constituents may contribute to reducing oxidative stress associated with T2DM.

Furthermore, AcEE demonstrated significant in vitro inhibitory activity against α-amylase and α-glucosidase, highlighting its potential as a natural source of enzyme inhibitors involved in postprandial glucose regulation. Complementary computational analyses supported these findings by predicting favorable interactions between several AcEE constituents and these enzymes. In addition, network pharmacology suggested that AcEE constituents may modulate key molecular targets associated with insulin resistance, inflammation, and diabetic complications, including INSR, Akt-1, STAT3, EGFR, TNF-α, IL-1β, and IL-6. Collectively, these findings support the hypothesis that AcEE may exert antidiabetic effects through a multitarget mechanism involving digestive enzyme inhibition, attenuation of oxidative stress and inflammation, and modulation of insulin signaling pathways.

The preliminary MTT assay indicated low in vitro cytotoxicity under the tested experimental conditions, although these findings should not be interpreted as a comprehensive toxicological assessment. Accordingly, further in vitro and in vivo toxicological studies are required to establish the safety profile of AcEE. Given the limited information currently available on the antidiabetic properties of *A. cruciatus*, this study provides a scientific basis for future investigations. Nevertheless, the proposed molecular mechanisms remain predictive and require experimental validation, while additional in vivo pharmacological and toxicological studies are warranted to confirm the therapeutic potential of *A. cruciatus* for the management of T2DM.

## Figures and Tables

**Table 1:** Extraction yield and total phenolic and flavonoid contents in the aerial parts of *Astragalus cruciatus*.

Sample	Extraction Yield (%)	Total Phenolic Content (mg GAE/g DW)	Total Flavonoid Content (mg QE/g DW)
AcEE	13.50	186.00 ± 0.20	45.63 ± 0.04

Results are expressed as mean ± SD, *n* = 3.

**Table 2:** Chemical composition of *Astragalus cruciatus* Link ethanolic extract.

Compound	Concentration (µg/g Extract)	Retention Time	*m*/*z*	Area	Ion Mod
Quinic acid	797.186	2.048	191.00	15,347,316	ESI (−)
Protocatechuic acid	41.435	6.910	153.00	53,729	ESI (−)
4-O-Caffeoylquinic acid	6.394	11.568	353.00	32,288	ESI (−)
Caffeic acid	0.909	14.464	353.00	6586	ESI (−)
Syringic acid	7.313	15.940	197.00	22,732	ESI (−)
p-Coumaric acid	66.329	20.793	163.00	413,188	ESI (−)
trans-Ferulic acid	23.780	22.940	193.00	176,210	ESI (−)
Hyperoside (quercetin-3-O-galactoside)	47.003	24.275	463.00	1,152,144	ESI (−)
Rutin	953.542	23.467	609.00	7,364,668	ESI (−)
O-Coumaric acid	4.558	26.313	163.00	18,986	ESI (−)
Apegenin-7-O-glucoside	2689	26,498	431,00	137,868	ESI (−)
Rosmarinic acid	3.408	26.638	359.00	35,336	ESI (−)
trans-cinnamic acid	38.401	31.806	147.00	9538	ESI (−)
Kaempherol	1.438	31.817	285.00	112,306	ESI (−)
Naringenin	0.758	33.752	271.00	45,496	ESI (−)
Apigenin	0.327	34.390	269.00	23,124	ESI (−)
Luteolin	2.170	34.728	285.00	64,106	ESI (−)
Cirsiliol	81.845	35.512	329.00	2,124,624	ESI (−)
Cirsilineol	5.495	38.506	343.00	354,801	ESI (−)

**Table 3:** Antioxidant potential of *Astragalus cruciatus* Link ethanolic extract.

Sample/ Standard	β-Carotene Bleaching	FIC	DPPH	FRAP	TAC
	IC_50_ (mg/mL)				mg GAE/g DW
AcEE	1.849 ± 0.017 ^b^	1.382 ± 0.017 ^b^	0.062 ± 0.003 ^b^	0.267 ± 0.012 ^b^	26.48 ± 0.38
BHA	0.267 ± 0.014 ^a^	-	0.032 ± 0.002 ^a^	0.100 ± 0.007 ^a^	-
EDTA	-	0.051 ± 0.001 ^a^	-	-	-

Results are presented as mean ± SD, *n* = 3. BHA, Butylated hydroxyanisole. EDTA, Ethylenediaminetetraacetic acid. Within each column, values with different letters (a,b) have statistical difference at *p* < 0.05.

**Table 4:** In vitro α-amylase and α-glucosidase inhibitory potential of AcEE compared to reference inhibitor.

Sample/ Standard	IC_50_ (µg/mL)	
	α-Amylase	α-Glucosidase
AcEE	239.3 ± 7.40 ^b^	192.60 ± 15.51 ^b^
Acarbose (reference drug)	94.78 ± 8.48 ^a^	211.70 ± 17.09 ^a^

Results are presented as mean ± SD, *n* = 3. Within each column, values with different letters have statistical difference. (a) significantly different from AcEE. (b) significantly different from acarbose at *p* < 0.001.

**Table 5:** The cytotoxic effect of AcEE and cisplatin on AML12 and HepG2 cell lines.

Sample/ Standard	IC_50_ (µg/mL)	
	AML12 Cell Line	HepG2 Cell Line
AcEE	403.40 ± 23.26 ^b^	396.30 ± 44.33 ^b^
Cisplatin (control)	8.34 ± 0.42 ^a^	5.95 ± 0.60 ^a^

Results are presented as mean ± SD, *n* = 3. (a) significantly different from AcEE. (b) significantly different from cisplatin at *p* < 0.001.

**Table 6:** Docking scores and binding free energies of AcEE constituents against α-amylase and α-glucosidase.

Compounds	α-Amylase (PDB ID: 1OSE)		α-Glucosidase (PDB ID: 3A4A)	
	Docking Scores (Kcal/mol)	MMGBSA (Kcal/mol)	Docking Scores (Kcal/mol)	MMGBSA (Kcal/mol)
Rutin	−12.935	−68.53	−8.073	−54.80
Acarbose (reference inhbitor)	−9.364	−62.02	−8.902	−58.60
Luteolin	−7.720	−51.92	−6.496	−41.54
Quercetin-3-O-galactoside	−7.607	−59.63	−7.627	−39.86
Kaempferol	−7.525	−49.57	−6.947	−39.40
Apigenin	−7.295	−50.40	−5.560	−52.06
Naringenin	−5.883	−45.60	−5.664	−50.62
Apegenin-7-O-glucoside	−5.623	−45.30	−6.895	−52.04
4-O-caffeoylquinic acid	−5.587	−40.91	−5.893	−33.04
Rosmarinic acid	−5.581	−36.10	−5.544	−31.60
O-Coumaric acid	−5.267	−22.13	−3.393	−18.94
Cirsiliol	−5.223	−35.60	−5.432	−30.21
Cirsilineol	−5.156	−32.24	−5.012	−33.60
Caffeic acid	−5.123	−10.43	−3.618	−14.50
Quinic acid	−4.648	−10.08	−5.147	−10.75
Ferulic acid	−4.152	−9.96	−3.814	−17.38
Trans-Cinnamic acid	−4.045	−18.98	−2.588	−13.53
Syringic acid	−3.830	−12.36	−3.296	−6.90
P-Coumaric acid	−3.640	−11.98	−3.664	−13.45
Protocatechuic acid	−3.631	−7.86	−4.660	−5.77

## Data Availability

The data presented in this study are available on request from the corresponding author.

## Supplementary Materials

The following supporting information can be downloaded at: https://www.mdpi.com/article/10.3390/molecules31162831/s1, Figure S1: Effects of different concentrations of AcEE on HepG2 (A), and AML12 (B) cell viability after 72 h of the treatment. Data are presented as mean ± S.D. * *p* < 0.05; ** *p* < 0.01; *** *p* < 0.001. *p*-value of less than 0.05 was considered statistically significant; Figure S2: Superimposition of the docked co-crystal ligand (in red color) and the co-crystal ligand (in green color) with RMSD value of 0.638 Å; Figure S3: 2D representation of molecular interactions of rutin with α-amylase active site; Figure S4: 2D representation of molecular interactions of acarbose in complex with α-amylase; Figure S5: 2D diagram of molecular interactions of rutin in complex with α-glucosidase active site; Figure S6: 2D diagram of molecular interactions of acarbose in complex with α-glucosidase active site; Figure S7: 2D interactions diagram of induced fit docking of: (A) rutin-α-amylase complex; (B) rutin-α-glucosidase complex; (C) acarbose-α-amylase; (D) acarbose-α-glucosidase; Figure S8: Protein–protein interaction network of common targets derived from STRING database; Figure S9: Topological analysis of the PPI network and hub genes; (A) degree, (B) closeness, (C) betweenness, (D) EPC, (E) MNC, (F) hub genes based on metrics; Figure S10: 2D interaction diagram of ACEE top binders with hub genes; (A) rutin-INSR complex, (B) rutin-EGFR complex, (C) luteolin-Akt-1 complex, (D) quercetin-3-O-galactoside-STAT3 complex, (E) quercetin-3-O-galactoside-TNF-α complex, (F) rosmarinic acid-IL1-β complex, (G) rosmarinic acid-IL-6 complex, (H) illustration; Figure S11: 2D interaction diagram of reference inhibitors with hub genes; (A) 60O-INSR complex, (B) 634-EGFR complex, (C) MK2206-Akt-1 complex, (D) SI109 -STAT3 complex, (E) SPD304 -TNF-α complex, (F) T9C-IL-1β complex, (G) LMT-28-IL-6 complex; Table S1: Validation of the docking procedure (RMSD calculation) and molecular docking results of the hub proteins and their corresponding reference inhibitors.
