# Supplementary material for: Phytochemical Profiling, In Vitro Bioactivity, and Network Pharmacology of Astragalus cruciatus Link Ethanolic Extract: Multitarget Mechanisms Underlying Potential Antidiabetic Effects
Source: Molecules. 2026 Aug 13;31(16):2831. doi: 10.3390/molecules31162831 (PMC13515701; doi:10.3390/molecules31162831)
Supplement: Supplementary file 1 [file molecules-31-02831-s001.zip › Supplementary data.pdf]

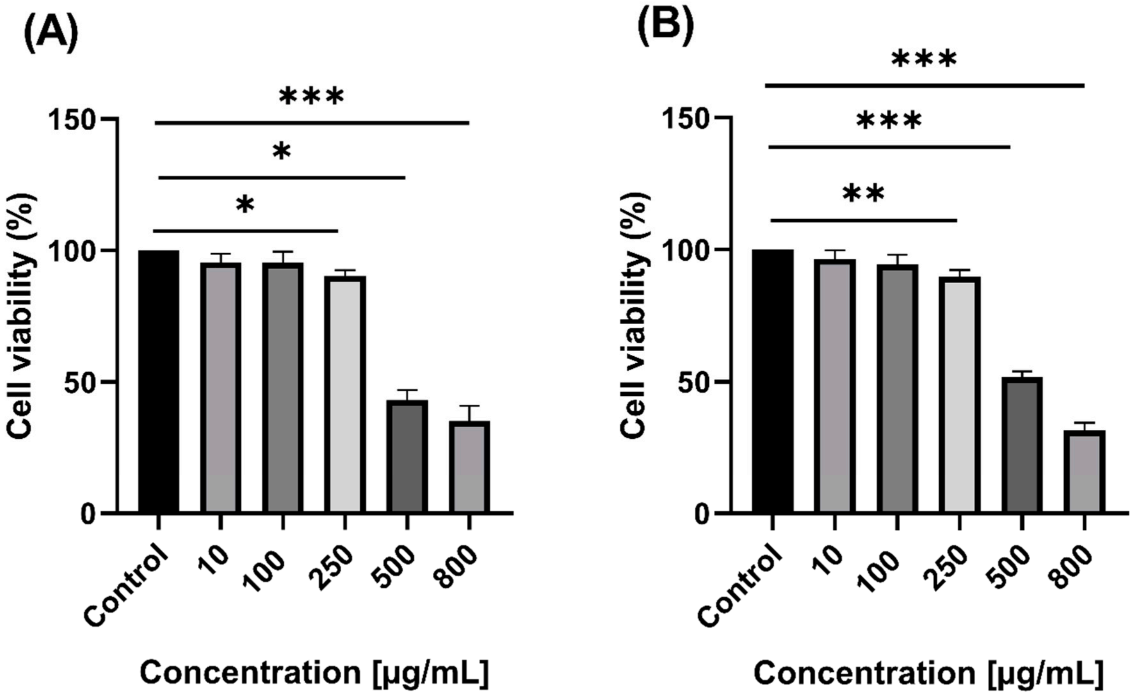

Figure S1. Effects of different concentrations of AcEE on HepG2 (**A**), and AML12 (**C**) cell viability after 72 h of the treatment. Data are presented as mean ± S.D. * $p < 0.05$; ** $p < 0.01$; ***$p < 0.001$. p-value of less than 0.05 was considered statistically significant.

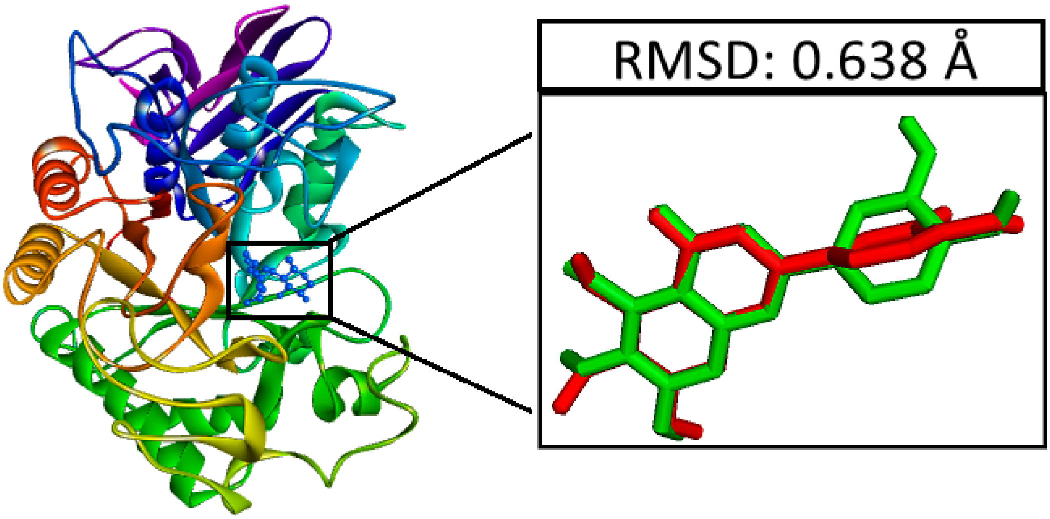

Figure S2. Superimposition of the docked co-crystal ligand (in red color) and the co-crystal ligand (in green color) with RMSD value of 0.638 Å.

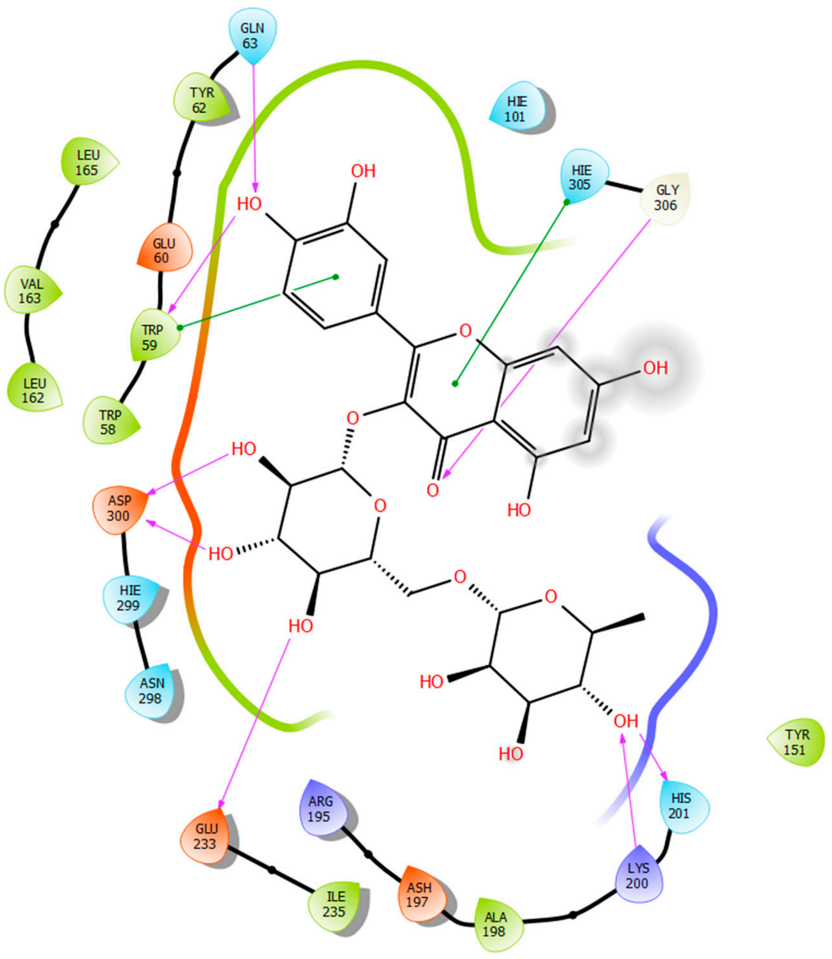

Figure S3. 2D representation of molecular interactions of rutin with α-amylase active site.

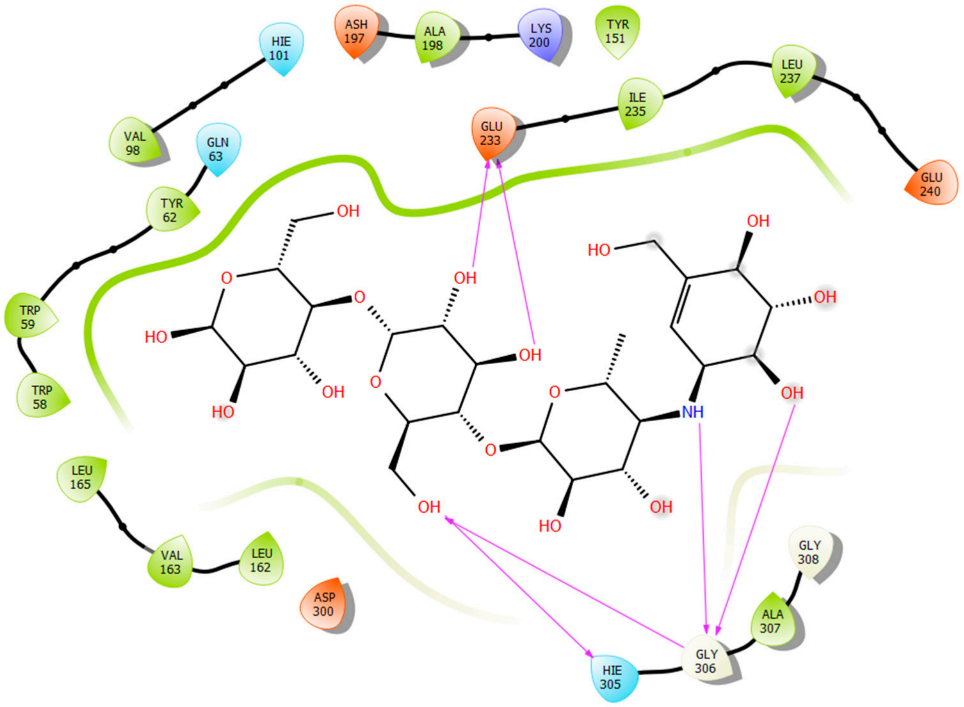

Figure S4. 2D representation of molecular interactions of acarbose in complex with α-amylase.

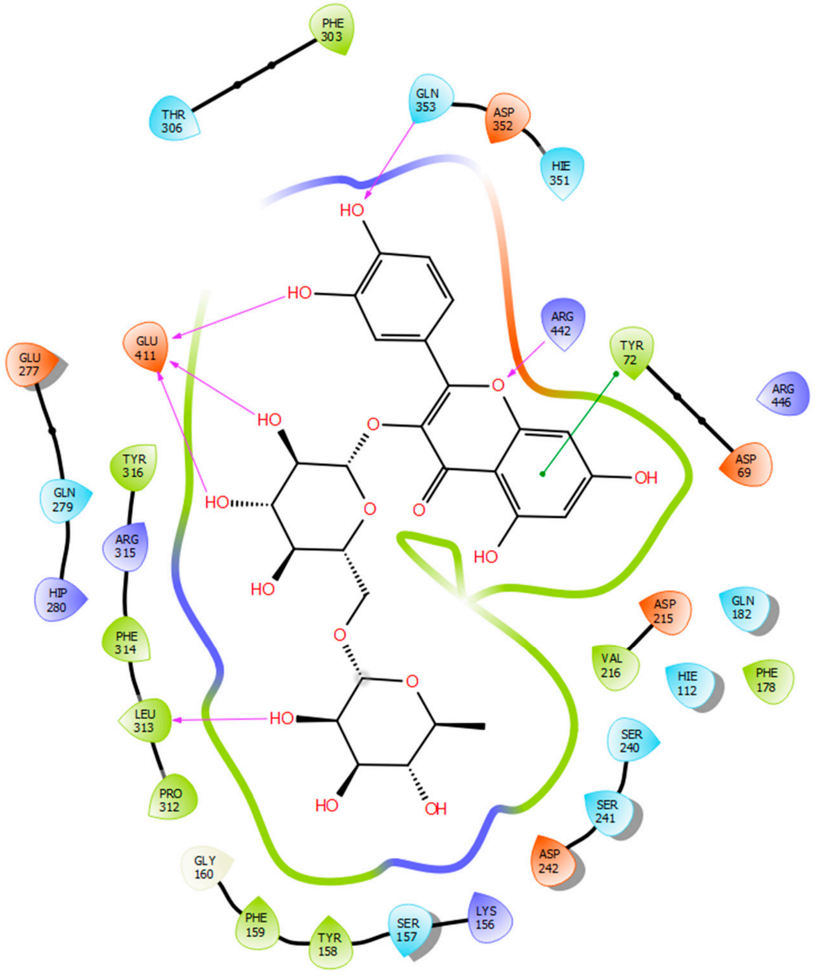

Figure S5. 2D diagram of molecular interactions of rutin in complex with α-glucosidase active site.

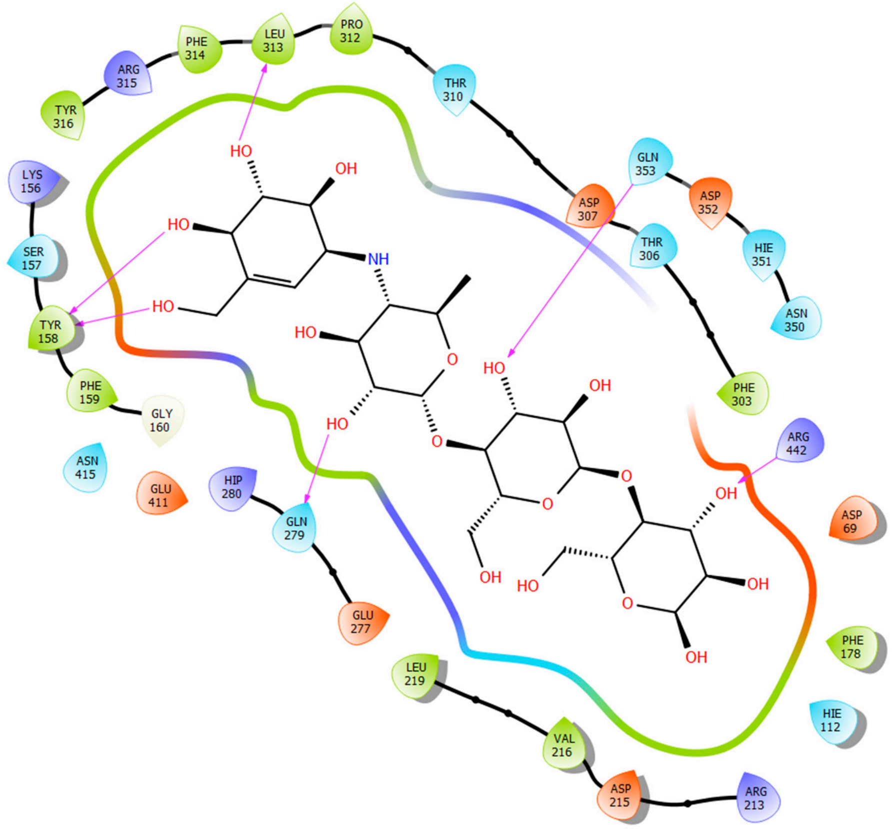

Figure S6. 2D diagram of molecular interactions of acarbose in complex with α-glucosidase active site.

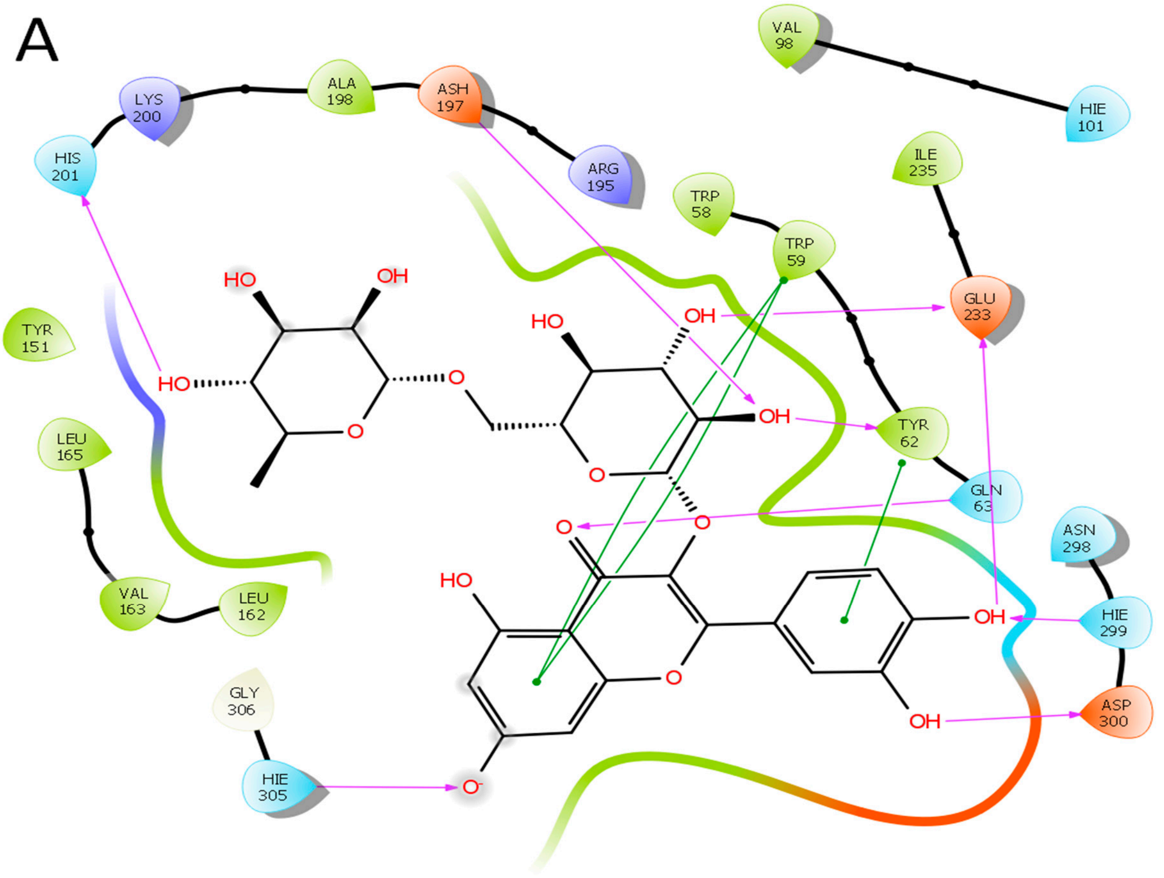

B

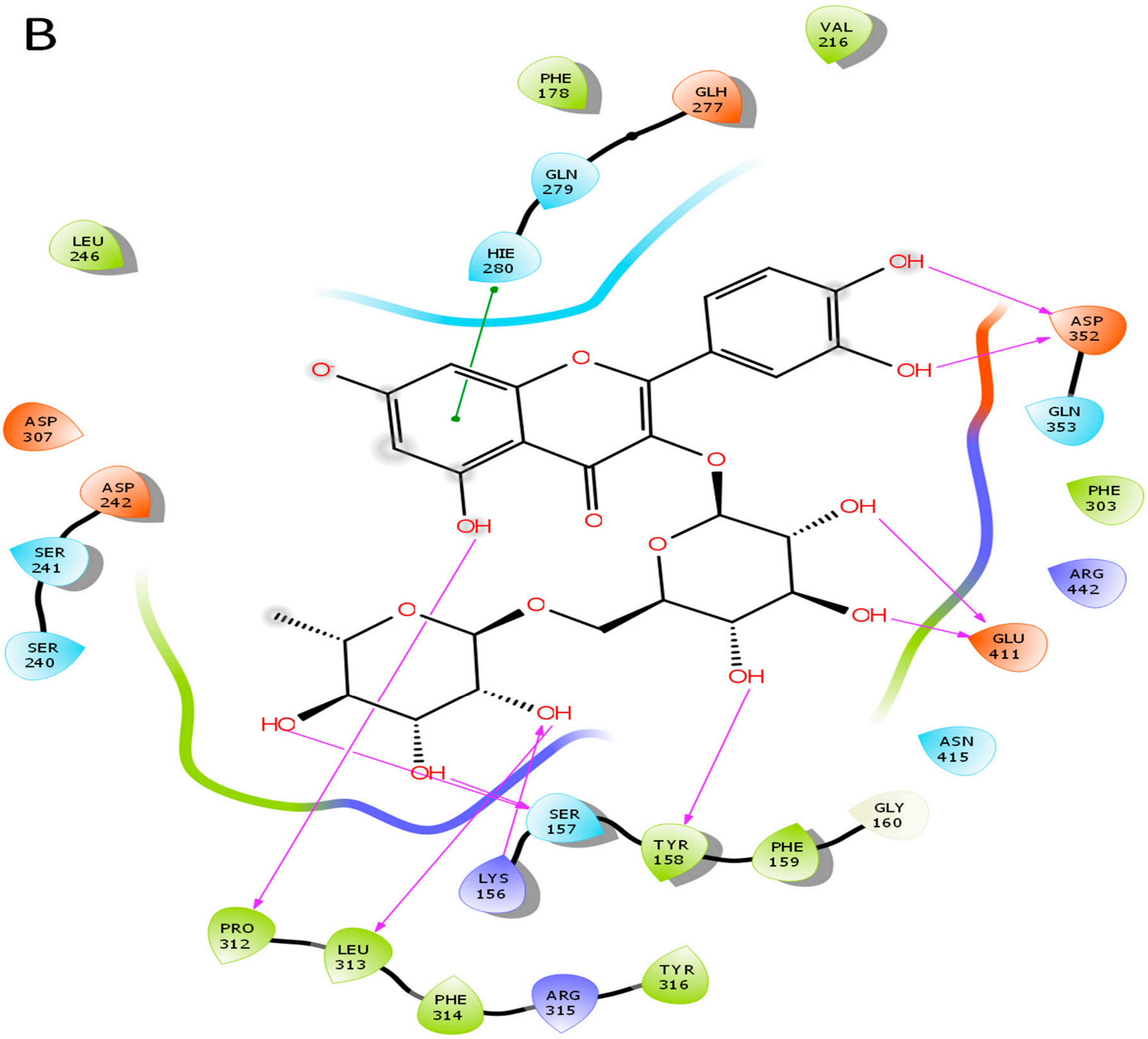
VAL 216
PHE 178
GLH 277
GLN 279
HIE 280
LEU 246
ASP 352
GLN 353
PHE 303
ARG 442
GLU 411
ASN 415
ASP 307
ASP 242
SER 241
SER 240
SER 157
TYR 158
PHE 159
GLY 160
LYS 156
PRO 312
LEU 313
PHE 314
ARG 315
TYR 316
OH
HO
O

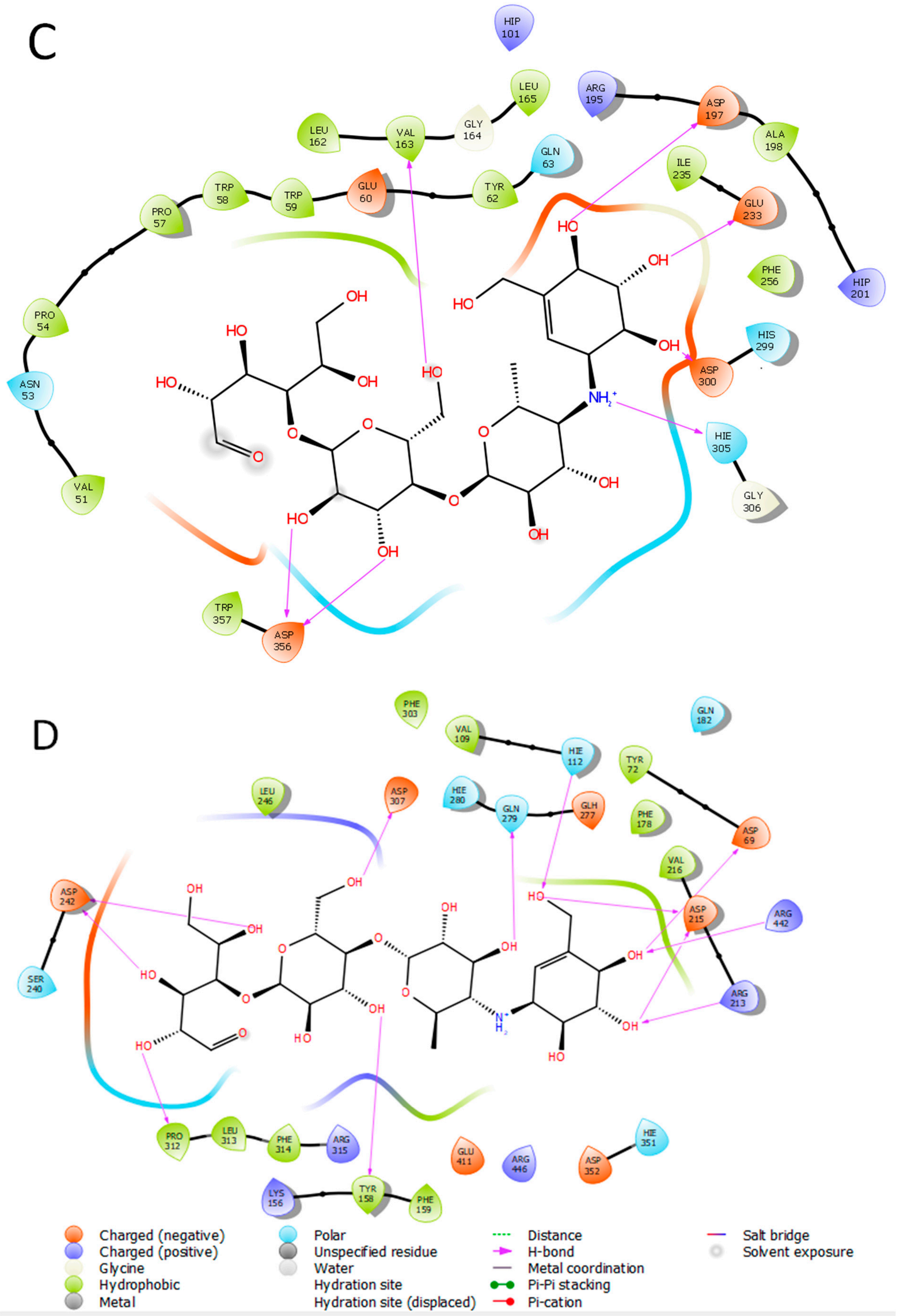
C
HIP 101
LEU 165
ARG 195
ASP 197
ALA 198
LEU 162
VAL 163
GLY 164
GLN 63
ILE 235
TRP 58
TRP 59
GLU 60
TYR 62
GLU 233
PRO 57
PHE 256
HIP 201
PRO 54
HIS 299
ASP 300
ASN 53
HIE 305
GLY 306
VAL 51
TRP 357
ASP 356
D
PHE 303
VAL 109
GLN 182
HIE 112
TYR 72
LEU 246
ASP 307
HIE 280
GLN 279
GLH 277
PHE 178
ASP 69
VAL 216
ASP 242
ASP 215
ARG 442
SER 240
ARG 213
PRO 312
LEU 313
PHE 314
ARG 315
GLU 411
ARG 446
ASP 352
HIE 351
LYS 156
TYR 158
PHE 159
Charged (negative)
Charged (positive)
Glycine
Hydrophobic
Metal
Polar
Unspecified residue
Water
Hydration site
Hydration site (displaced)
Distance
H-bond
Metal coordination
Pi-Pi stacking
Pi-cation
Salt bridge
Solvent exposure

Figure S7. 2D interactions diagram of induced fit docking of: (A) rutin-α-amylase complex; (B) rutin-α-glucosidase complex; (C) acarbose-α-amylase; (D) acarbose-α-glucosidase.

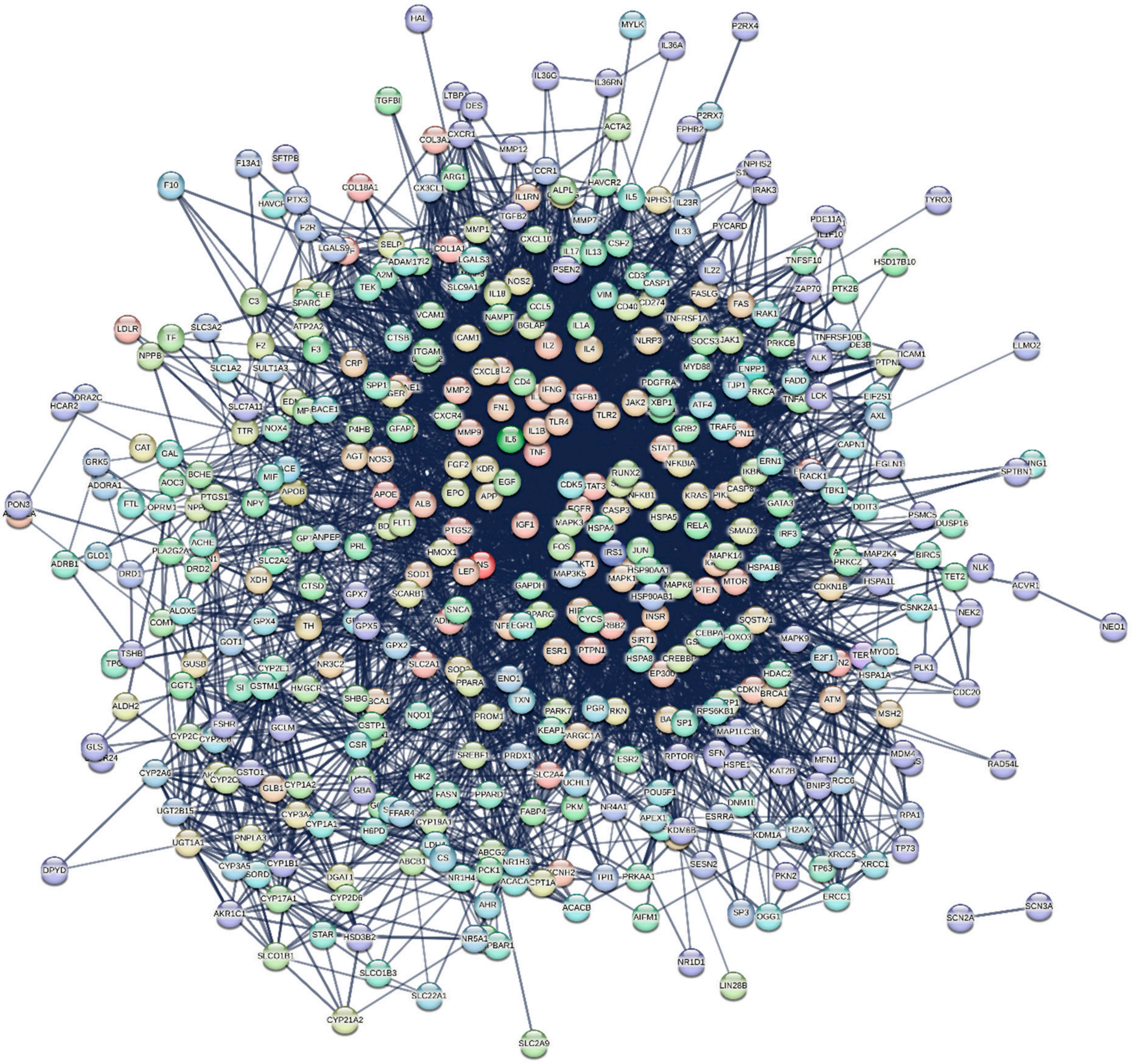

Figure S8. Protein-protein interaction network of common targets derived from STRING database.

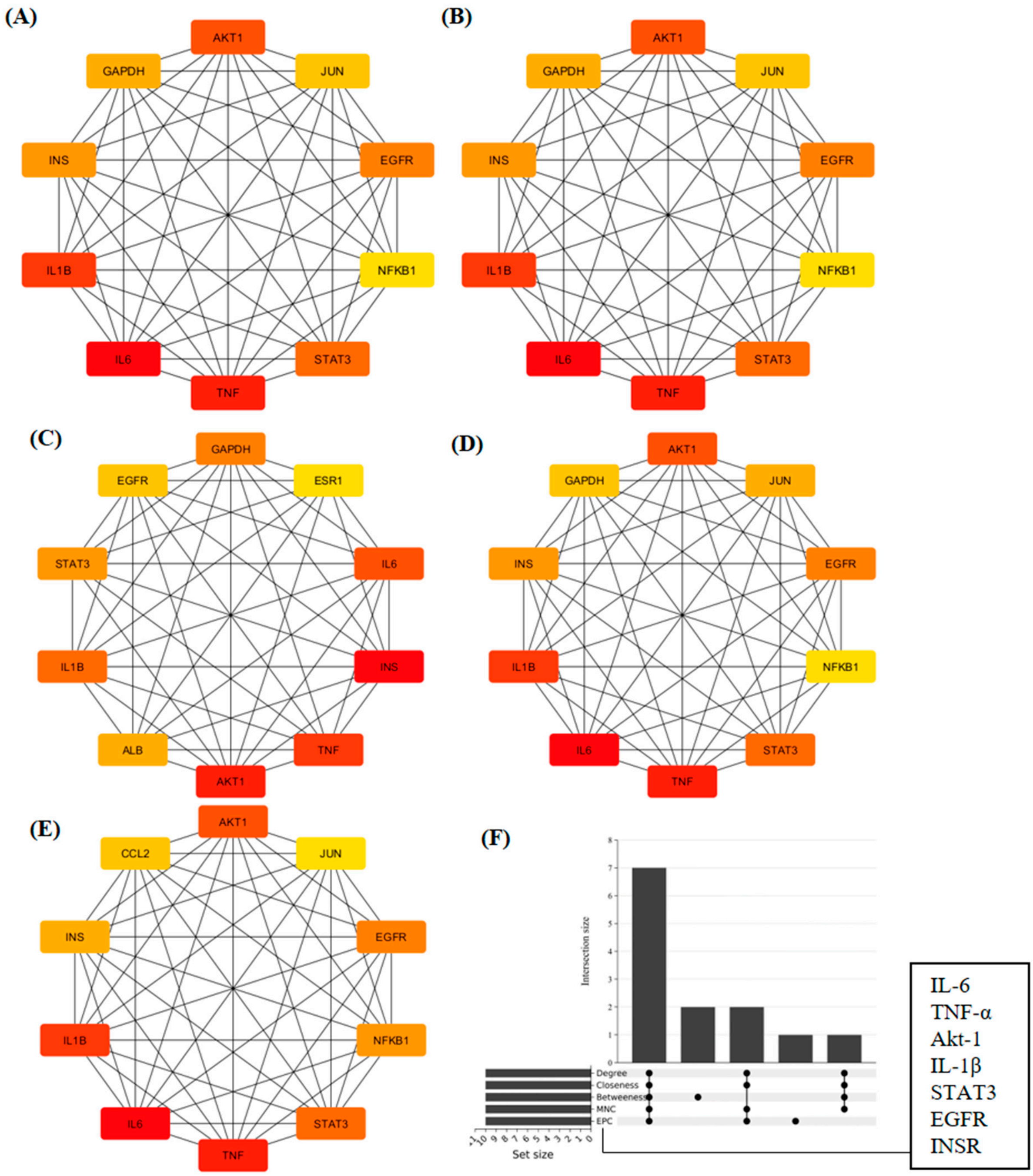

Figure S9. Topological analysis of the PPI network and hub genes; (A) degree, (B) closeness, (C) betweeness, (D) EPC, (E) MNC, (F) hub genes based on metrics.

(A)

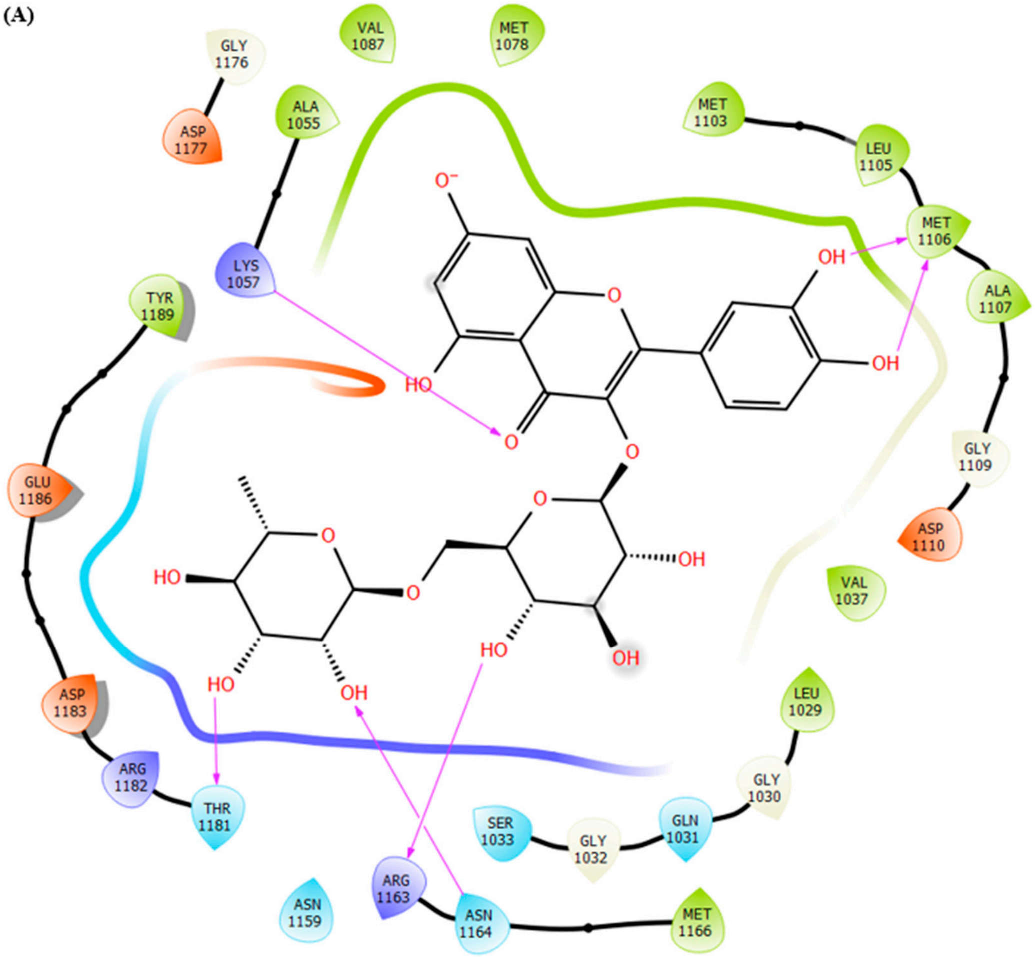

GLY 1176
VAL 1087
MET 1078
ASP 1177
ALA 1055
MET 1103
LEU 1105
MET 1106
LYS 1057
TYR 1189
ALA 1107
GLY 1109
GLU 1186
ASP 1110
VAL 1037
LEU 1029
ASP 1183
GLY 1030
ARG 1182
THR 1181
SER 1033
GLY 1032
GLN 1031
ASN 1159
ARG 1163
ASN 1164
MET 1166

Continued.

(B)

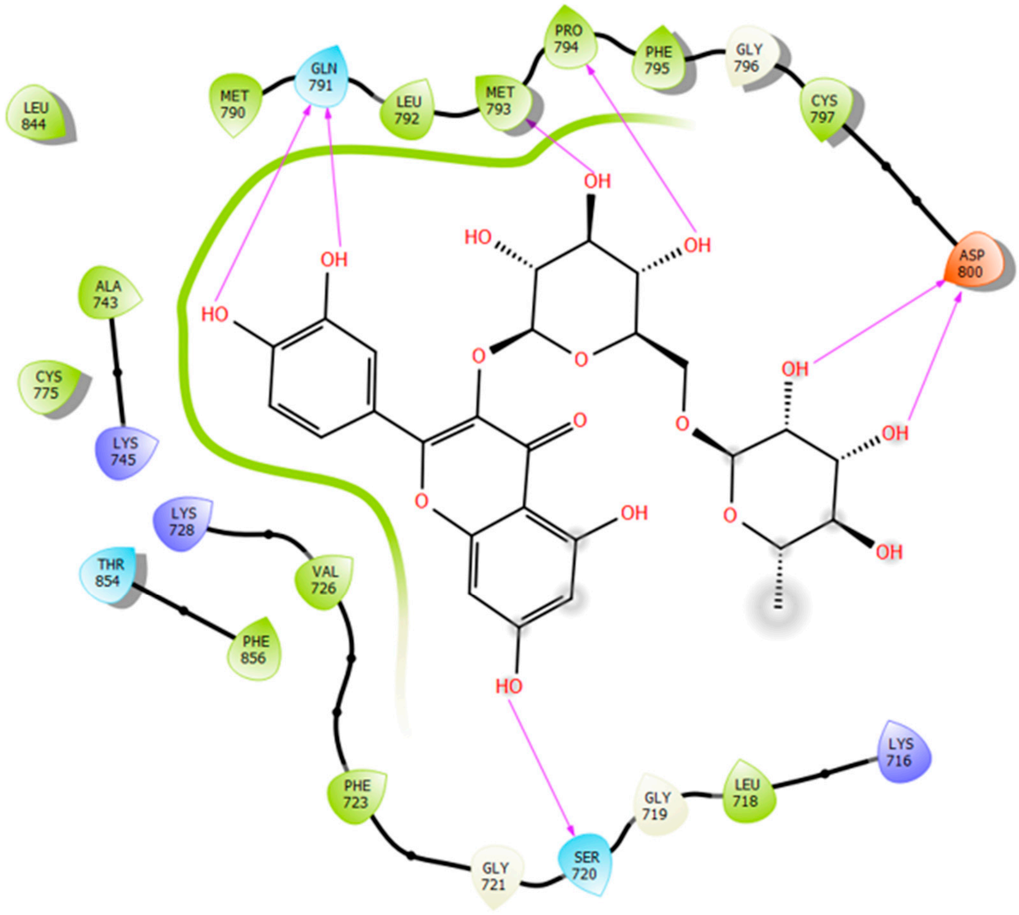

LEU 844
MET 790
GLN 791
LEU 792
MET 793
PRO 794
PHE 795
GLY 796
CYS 797
ASP 800
ALA 743
CYS 775
LYS 745
LYS 728
THR 854
VAL 726
PHE 856
PHE 723
GLY 721
SER 720
GLY 719
LEU 718
LYS 716
OH
HO
O

Continued.

(C)

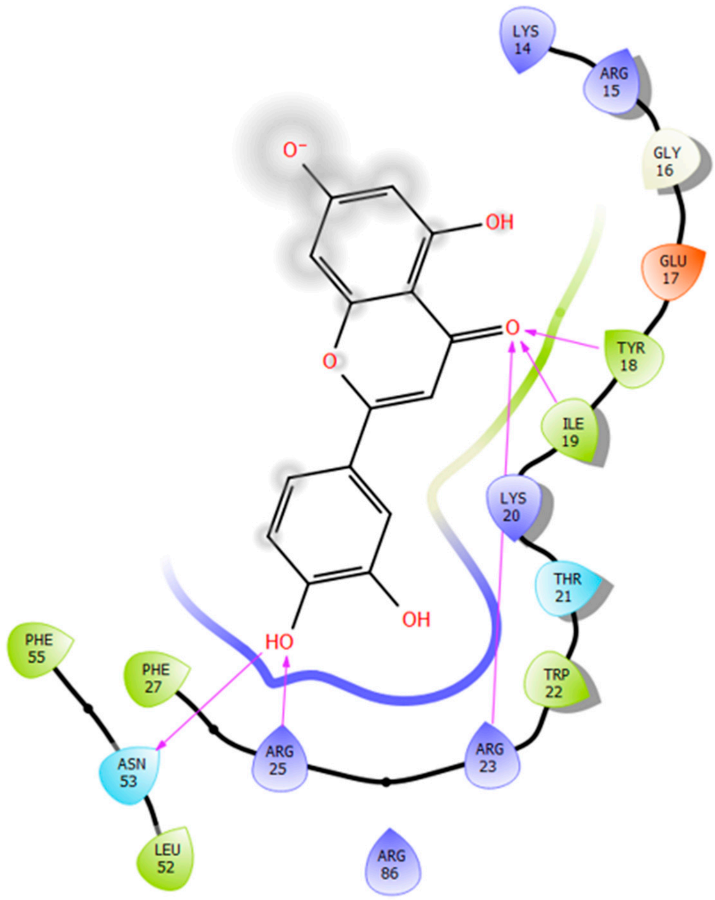
LYS 14
ARG 15
GLY 16
GLU 17
TYR 18
ILE 19
LYS 20
THR 21
TRP 22
ARG 23
ARG 25
PHE 27
PHE 55
ASN 53
LEU 52
ARG 86
O⁻
OH
O
O
HO
OH

(D)

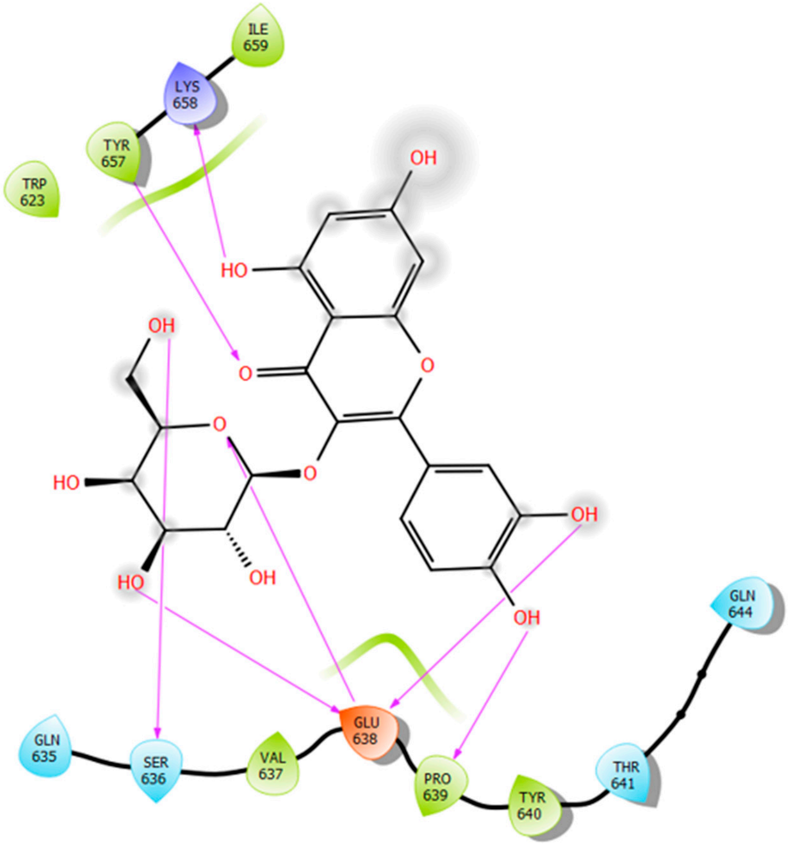
ILE 659
LYS 658
TYR 657
TRP 623
OH
HO
OH
O
O
O
HO
O
OH
HO
OH
OH
GLN 644
GLN 635
SER 636
VAL 637
GLU 638
PRO 639
TYR 640
THR 641

Continued.

(E)

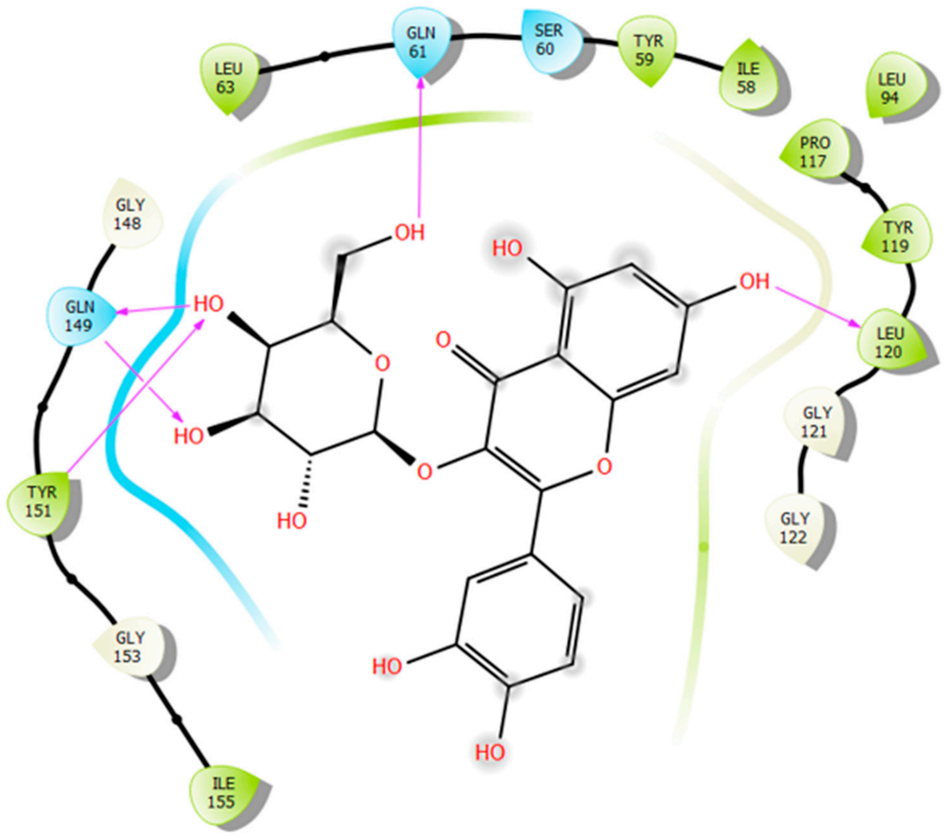

Continued.

(F)

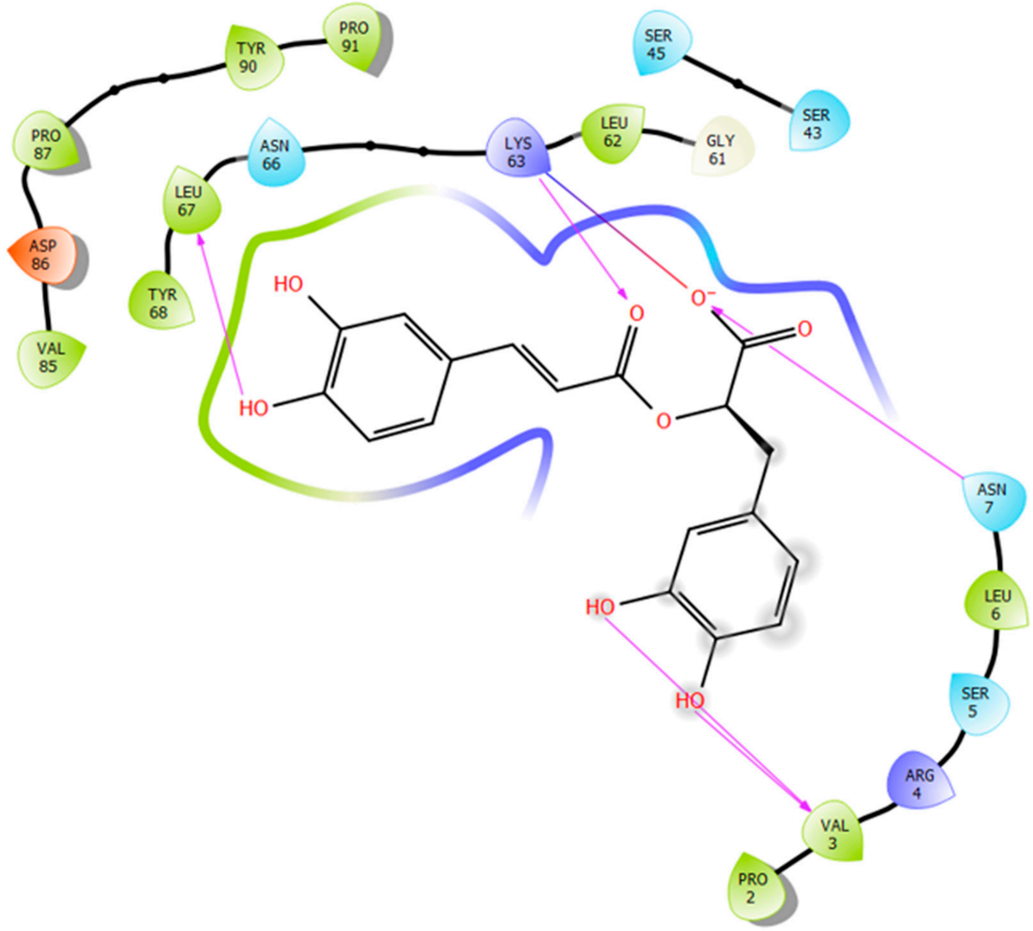
PRO 91
TYR 90
PRO 87
ASP 86
VAL 85
SER 45
SER 43
ASN 66
LYS 63
LEU 62
GLY 61
LEU 67
TYR 68
HO
HO
O
O
O⁻
O
ASN 7
LEU 6
SER 5
ARG 4
VAL 3
PRO 2
HO
HO

(G)

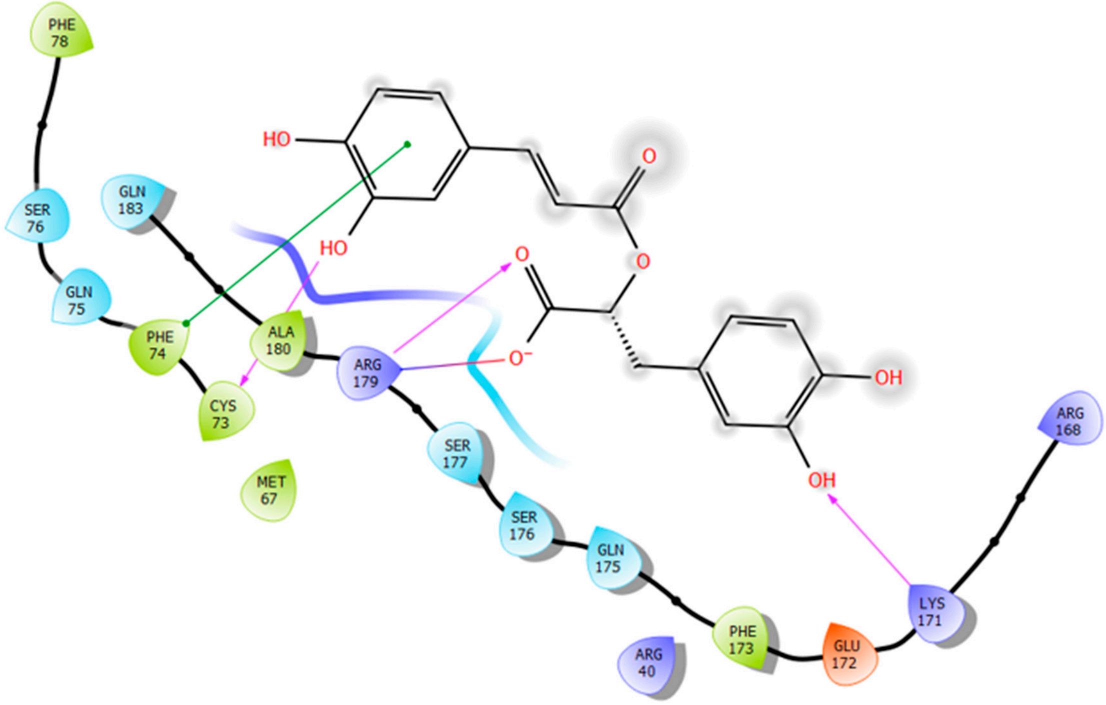
PHE 78
SER 76
GLN 75
GLN 183
PHE 74
ALA 180
CYS 73
MET 67
ARG 179
SER 177
SER 176
GLN 175
PHE 173
GLU 172
LYS 171
ARG 168
ARG 40
HO
HO
O
O
O
O⁻
OH
OH

(H)

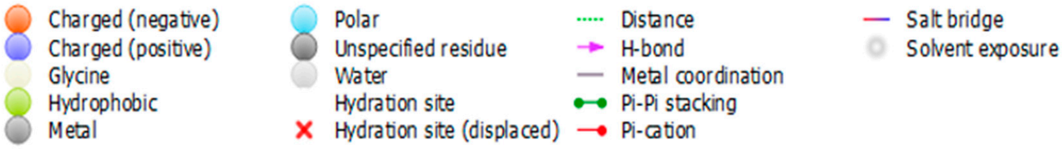

Figure S10. 2D interaction diagram of AcEE top binders with hub genes; (A) rutin-INSR complex, (B) rutin-EGFR complex, (C) luteolin-Akt-1 complex, (D) quercetin-3-O-galactoside-STAT3 complex, (E) quercetin-3-O-galactoside-TNF-α complex, (F) rosmarinic acid-IL-1β complex, (G) rosmarinic acid-IL-6 complex, (H) illustration.

(A)

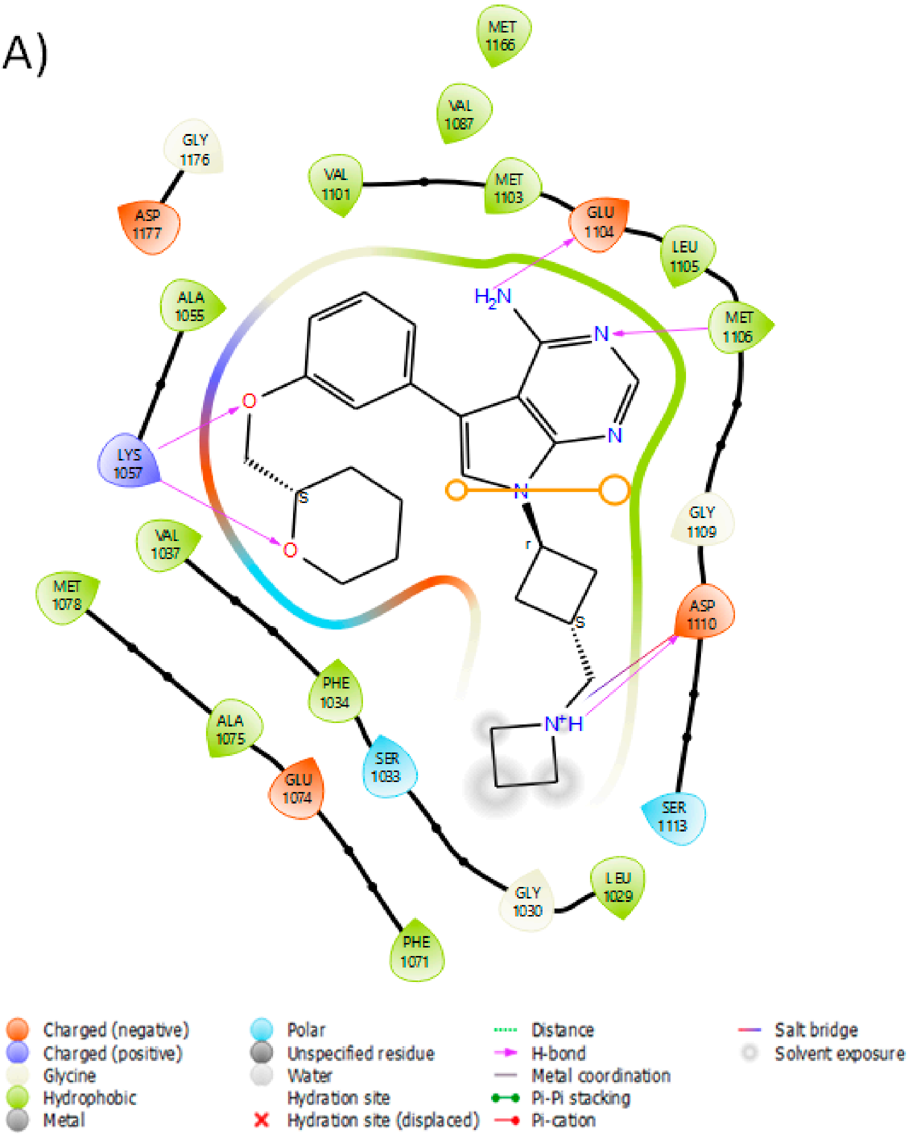

(B)

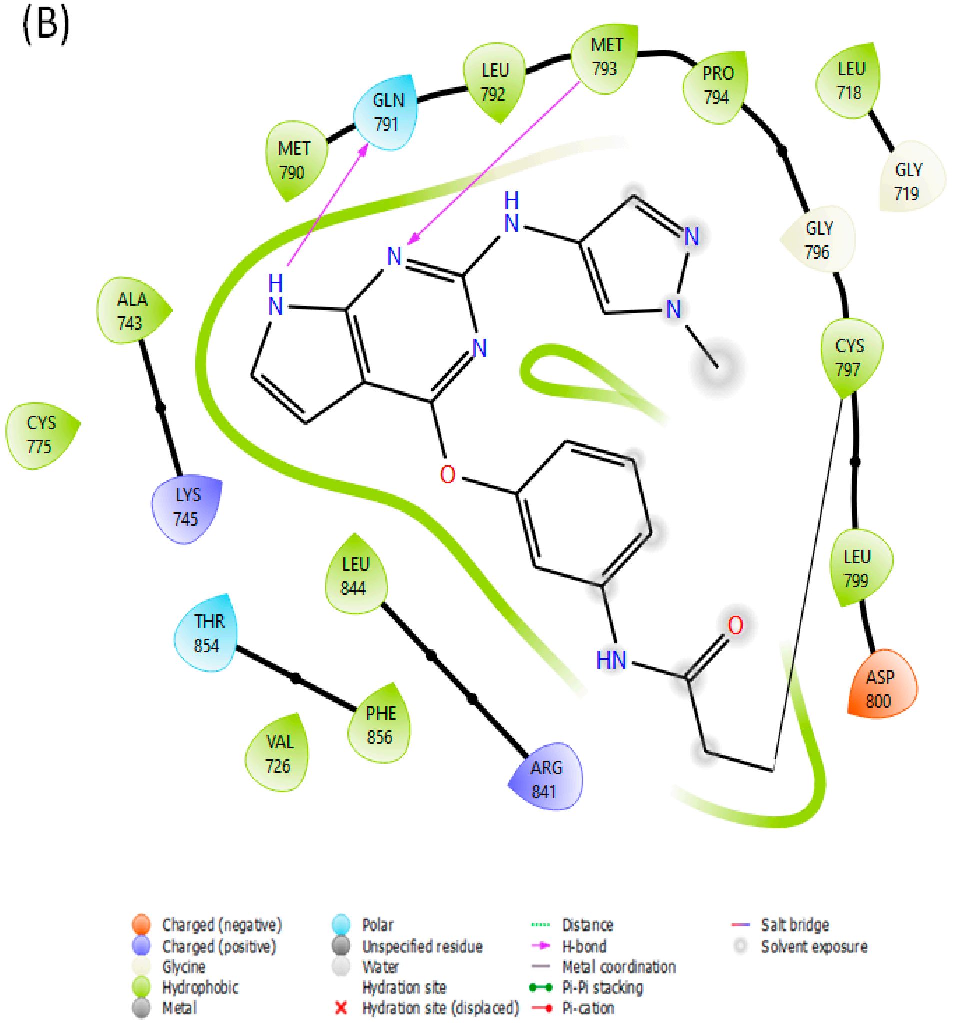

MET 790
GLN 791
LEU 792
MET 793
PRO 794
LEU 718
GLY 719
GLY 796
CYS 797
LEU 799
ASP 800
ALA 743
CYS 775
LYS 745
LEU 844
THR 854
PHE 856
VAL 726
ARG 841
H N
N
N
H N
N
N
O
HN
O
Charged (negative)
Charged (positive)
Glycine
Hydrophobic
Metal
Polar
Unspecified residue
Water
Hydration site
Hydration site (displaced)
Distance
H-bond
Metal coordination
Pi-Pi stacking
Pi-cation
Salt bridge
Solvent exposure

C)

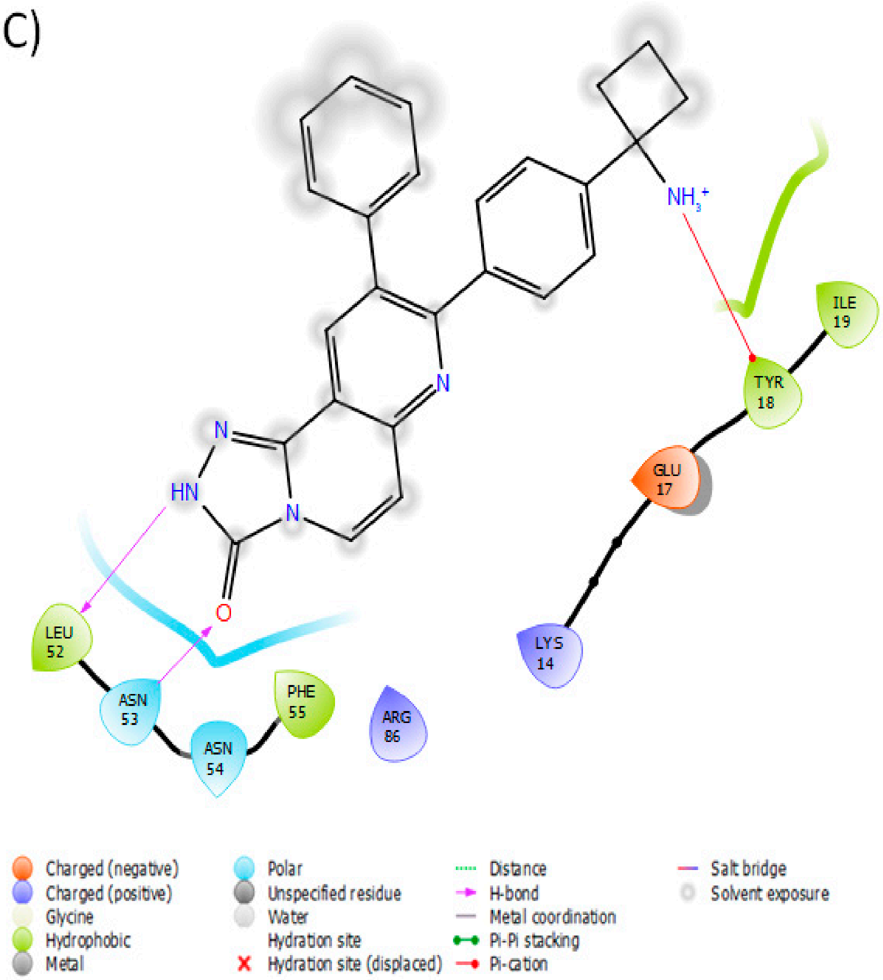

NH3+
N
HN
N
N
O
ILE 19
TYR 18
GLU 17
LYS 14
LEU 52
ASN 53
ASN 54
PHE 55
ARG 86
Charged (negative)
Charged (positive)
Glycine
Hydrophobic
Metal
Polar
Unspecified residue
Water
Hydration site
Hydration site (displaced)
Distance
H-bond
Metal coordination
Pi-Pi stacking
Pi-cation
Salt bridge
Solvent exposure

(D)

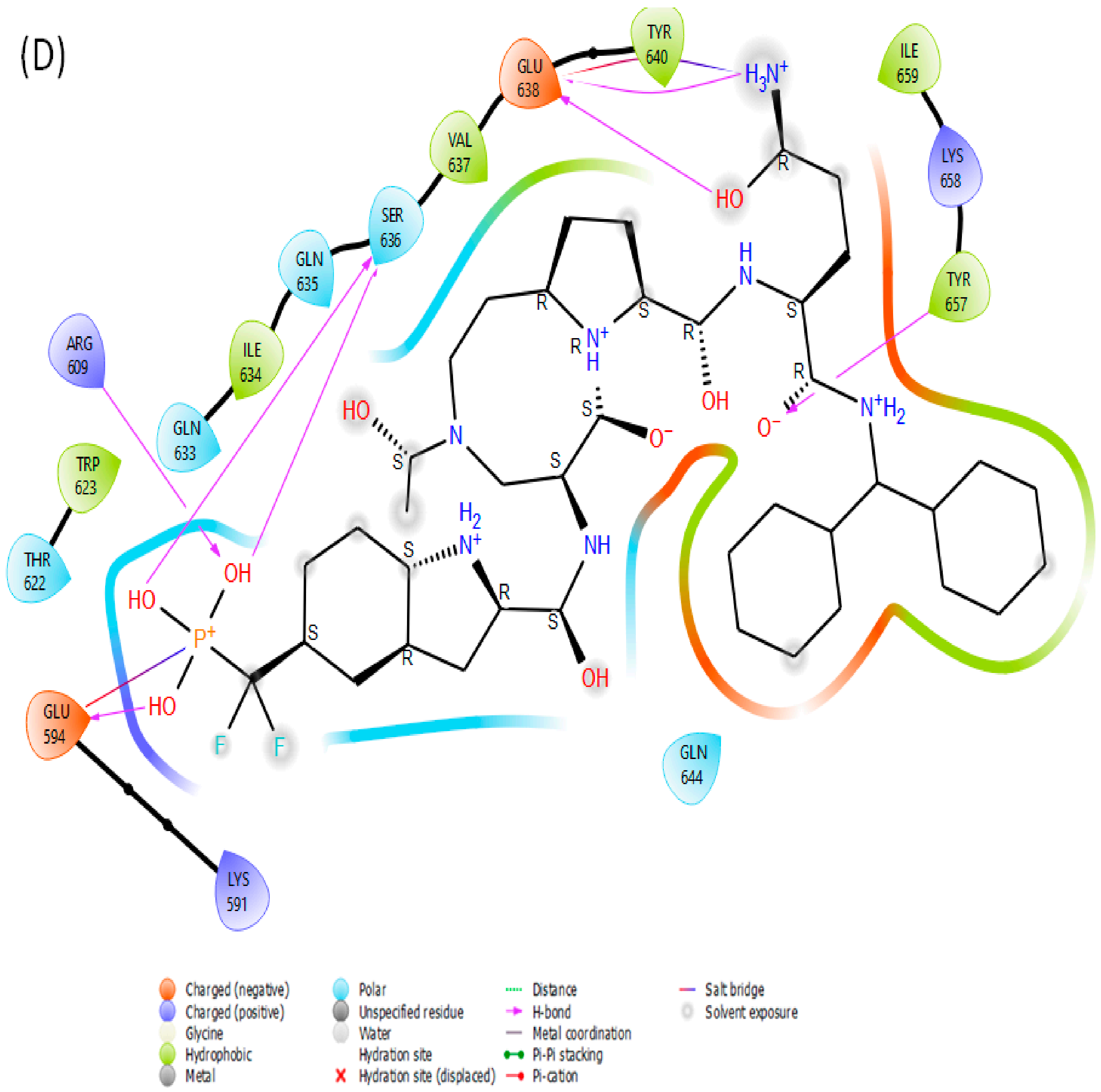

TYR 640
GLU 638
VAL 637
SER 636
GLN 635
ILE 634
GLN 633
ARG 609
TRP 623
THR 622
GLU 594
LYS 591
ILE 659
LYS 658
TYR 657
GLN 644
Charged (negative)
Charged (positive)
Glycine
Hydrophobic
Metal
Polar
Unspecified residue
Water
Hydration site
Hydration site (displaced)
Distance
H-bond
Metal coordination
Pi-Pi stacking
Pi-cation
Salt bridge
Solvent exposure

(E)

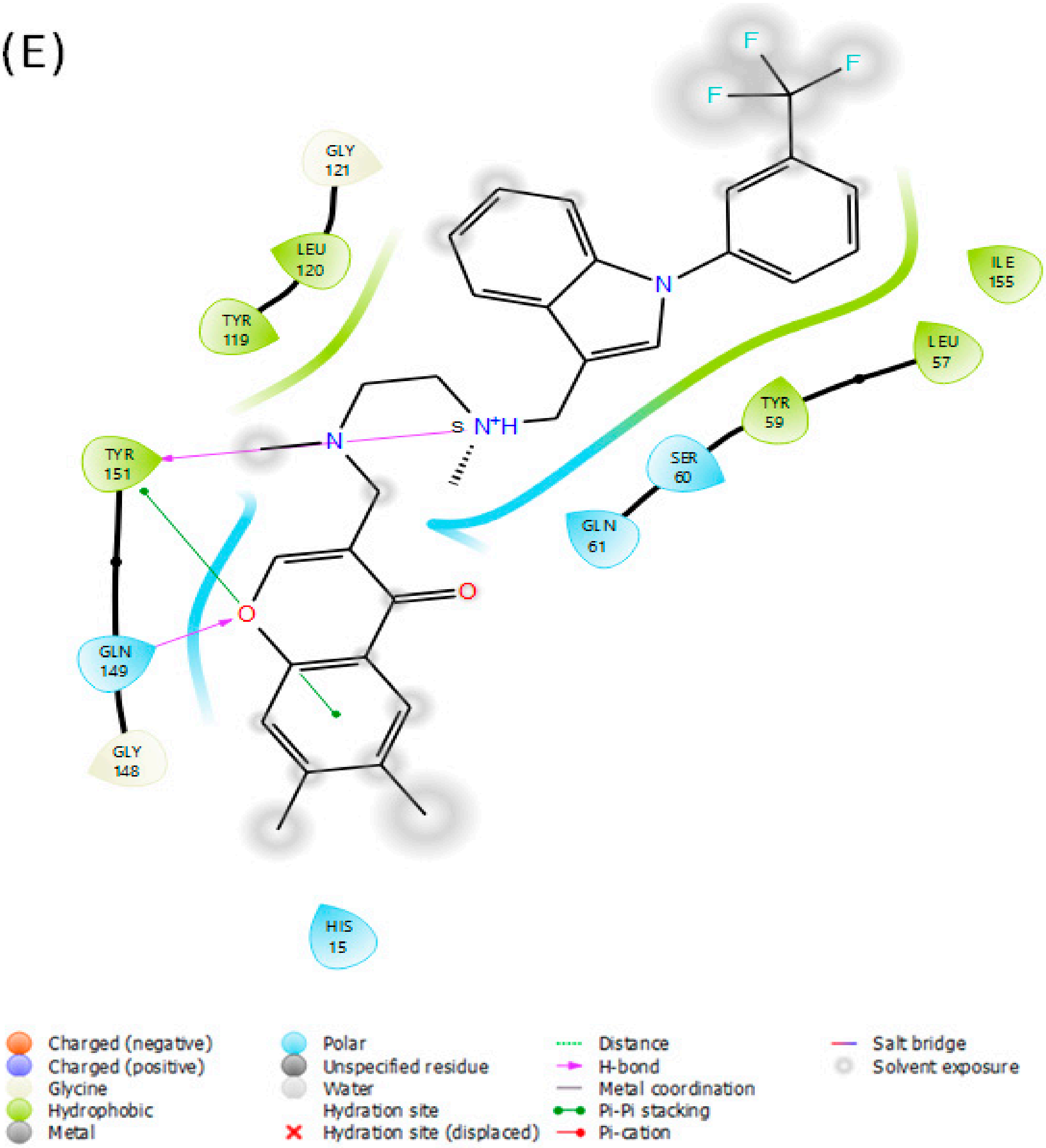
F
F
F
GLY 121
LEU 120
TYR 119
N
ILE 155
LEU 57
TYR 59
N
S
N⁺H
TYR 151
SER 60
GLN 61
O
O
GLN 149
GLY 148
HIS 15
Charged (negative)
Charged (positive)
Glycine
Hydrophobic
Metal
Polar
Unspecified residue
Water
Hydration site
Hydration site (displaced)
Distance
H-bond
Metal coordination
Pi-Pi stacking
Pi-cation
Salt bridge
Solvent exposure

(F)

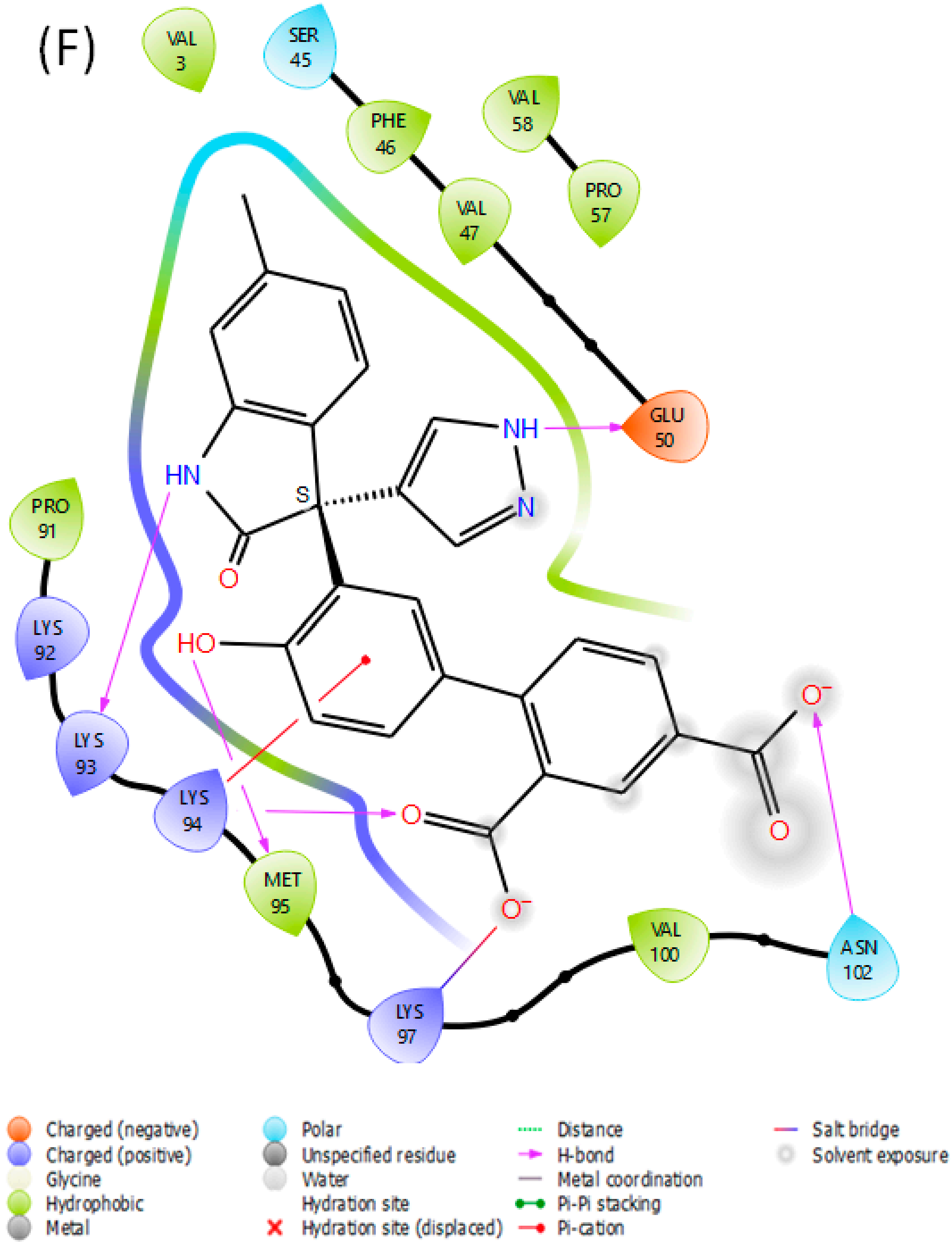

VAL 3
SER 45
PHE 46
VAL 58
PRO 57
VAL 47
GLU 50
NH
N
HN
S
O
PRO 91
LYS 92
HO
LYS 93
LYS 94
O
O⁻
O
O⁻
MET 95
VAL 100
ASN 102
LYS 97
Charged (negative)
Charged (positive)
Glycine
Hydrophobic
Metal
Polar
Unspecified residue
Water
Hydration site
Hydration site (displaced)
Distance
H-bond
Metal coordination
Pi-Pi stacking
Pi-cation
Salt bridge
Solvent exposure

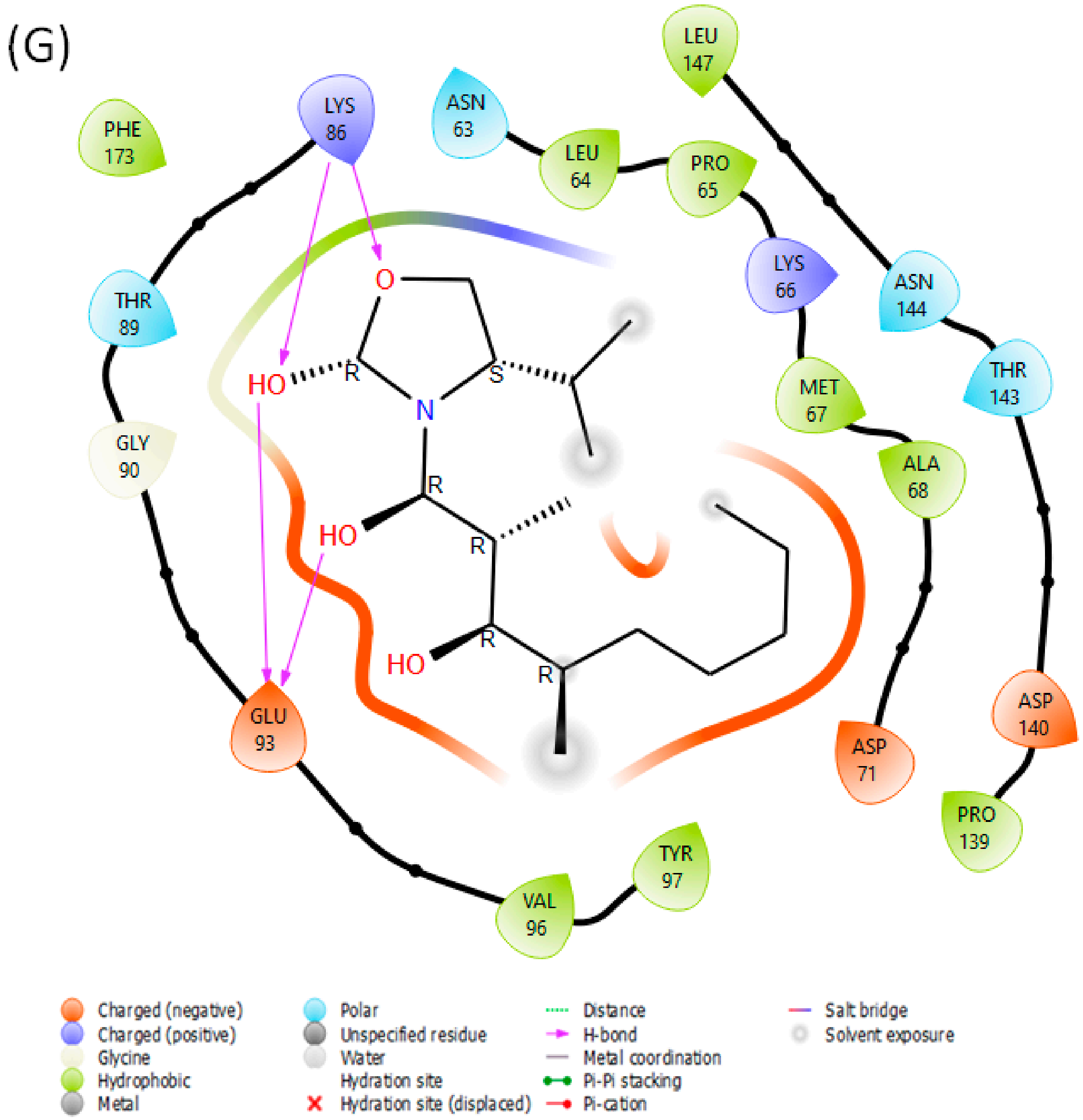

Figure S11. 2D interaction diagram of reference inhibitors with hub genes; (A) 60O-INSR complex, (B) 634-EGFR complex, (C) MK2206-Akt-1 complex, (D) SI109 -STAT3 complex, (E) SPD304 -TNF-α complex, (F) T9C-IL-1β complex, (G) LMT-28-IL-6 complex.
