# Supplementary material for: Phytochemical Profiling, In Vitro Bioactivity, and Network Pharmacology of Astragalus cruciatus Link Ethanolic Extract: Multitarget Mechanisms Underlying Potential Antidiabetic Effects
Source: Molecules. 2026 Aug 13;31(16):2831. doi: 10.3390/molecules31162831 (PMC13515701; doi:10.3390/molecules31162831)
Supplement: Supplementary file 1 [file molecules-31-02831-s001.zip › Table S1.pdf]

Table S1: Validation of the docking procedure (RMSD calculation) and molecular docking results of the hub proteins and their corresponding reference inhibitors.

Protein (PDB ID)	Reference inhibitor	Co-crystallized ligand	Docking scores (Kcal/mol)	RMSD (Å)	Interacting Residues of binding site
INSR (PDB ID: 5HHW)	7-[3-(azetidin-1-ylmethyl) cyclobutyl]-5-[3-[[(2~{R})-oxan-2-yl]methoxy]phenyl] pyrrolo[2,3-d]pyrimidin-4-amine.	7-[3-(azetidin-1-ylmethyl) cyclobutyl]-5-[3-[[(2~{R})-oxan-2-yl]methoxy]phenyl]pyrrolo[2,3-d]pyrimidin-4-amine.	-11.88	0.792	MET 1166, VAL1087, GLY1176, ASP1177, VAL1101, MET 1103, GLU1104, LEU1105, ALA1055, MET1106, LYS1057, GLY 1109, VAL1037, ASP1110, MET1078, PHE1034, AL A1075, SER 1033, GLU1074, SER1113, PHE1071, GLY1030, LEU1029.
EGFR (PDB ID: 5HG8)	N-[3-({2-[(1-methyl-1H-pyrazol-4-yl) amino]-7H-pyrrolo[2,3-d]pyrimidin-4-yl}oxy)phenyl]propanamide.	N-[3-({2-[(1-methyl-1H-pyrazol-4-yl) amino]-7H-pyrrolo[2,3-d]pyrimidin-4-yl}oxy)phenyl] propanamide.	-4.91	1.865	MET790, GLN791, LEU792, MET793, PRO794, LEU718, GLY719, GLY796, CYS797, ALA743, CYS775, LYS 745, LEU 799, ASP800, LEU844, THR854, VAL726, PHE856, ARG841.
AKT1 (PDB ID: 1UNQ)[a]	MK2206	/	-3.94	/	ILE19, TYR18, GLU17, LYS14, LEU52, ASN53, ASN54, PHE55, ARG86.
STAT3 (PDB ID: 6NUQ)	SI109	SI109	-6.74	1.953	GLU638, TYR640, ILE659, LYS658, TYR657, GLN644, LYS591, GLU594, THR622, TRP623, ARG609, GLN633, ILE634, GLN635, SER636, VAL637.
TNF-α (PDB ID: 2AZ5)	SPD304	SPD304	-3.33	1.177	GLY121, LEU120, TYR119, TYR151, GLN149, GLY148, HIS15, SER60, GLN61, TYR59, LEU57, ILE155.
IL-1β (PDB ID: 8RYK)[a]	T9C	/	-8.47	/	VAL3, SER45, PHE46, VAL47, VAL58, PRO57, GLU50, PRO91, LYS92, LYS93, LYS94, MET95, LYS97, VAL100, ASN102.
IL-6 (PDB ID: 5FUC)[a]	LMT-28	/	-4.15	/	PHE173, THR89, GLY90, LYS86, ASN63, LEU64, PRO65, LEU147, LYS66, ASN144, THR143, MET67, ALA68, ASP140, PRO139, ASP71, TYR97, VAL96, GLU93.

[a] Redocking validation was not performed for the receptors AKT1, IL1B, and IL6 because these receptors had no co-crystallized ligands. The docking score was calculated for their reference inhibitors for comparison with the identified AcEE compounds.
